# Flow Cytometry-Based Protocols for the Analysis of Human Plasma Cell Differentiation

**DOI:** 10.3389/fimmu.2020.571321

**Published:** 2020-09-29

**Authors:** Sharesta Khoenkhoen, Monika Ádori, Gabriel K. Pedersen, Gunilla B. Karlsson Hedestam

**Affiliations:** Department of Microbiology, Tumor and Cell Biology, Karolinska Institutet, Stockholm, Sweden

**Keywords:** plasma cell differentiation, Blimp-1/PRDM1, IRF4, Pax5, CVID, common variable immunodeficiency, B cell proliferation

## Abstract

Humoral immunity is established after differentiation of antigen-specific B cells into plasma cells (PCs) that produce antibodies of relevant specificities. Defects in the development, activation, or differentiation of B cells severely compromises the immune response. Primary immunodeficiencies are often characterized by hypogammaglobulinemia and the inability to mount effective antigen-specific antibody responses, resulting in increased susceptibility to infections. After IgA deficiency, which is most often asymptomatic, common variable immunodeficiency (CVID) is the most prevalent symptomatic primary immunodeficiency, but in most cases the underlying genetic causes are unknown or their roles in disease pathogenesis are poorly understood. In this study, we developed a protocol for *in vitro* stimulation of primary human B cells for subsequent analyses of PC differentiation and antibody production. With this approach, we were able to detect a population of CD38^+^ IRF4^+^ Blimp-1^+^ cells committed to PC fate and IgG production, including when starting from cryopreserved samples. The application of functional assays to characterize PC differentiation and possible defects therein in B cells from patients suffering from primary antibody deficiencies with late B cell defects could increase our understanding of the disease pathophysiology and underlying mechanisms.

## Introduction

The production of antibodies that prevent or limit infections is central to humoral immunity. Recognition of pathogens by B cells and their activation is achieved through B cell receptor (BCR) signaling. In addition, efficient activation and differentiation of B cells into effector PCs requires either engagement of Toll-like receptors (TLRs) that are able to sense pathogen-derived molecules, or ligation of costimulatory receptors that enable crosstalk with other immune cells. Additionally, cytokines interacting with their receptors expressed on B cells can enhance and/or modulate the response. A hallmark of B cell responses is the generation of germinal centers (GCs) in draining lymph nodes and the spleen. GCs are microenvironments where activated B cells receive signals from T follicular helper (Tfh) cells to undergo BCR affinity maturation and selection (1, 2). Tfh cells support B cell activation and differentiation through CD40L-CD40, CD28-CD80/86, and ICOS-ICOSL interactions, and through the production of cytokines, such as IL-2 and IL-4 that increase B cell proliferation, and IL-10 and IL-21, that enhance PC differentiation (3–8). Activated B cells may differentiate into either antibody-producing PCs or memory B cells, where the latter are programmed to rapidly differentiate into PCs after antigen re-exposure (9, 10).

Most of our understanding of PC differentiation and regulation has been acquired from mouse models, in which genetic defects or engineered reporter systems have provided detailed insight in the regulation of the PC lineage. The process of differentiation and fate commitment is an intricately regulated process that involves the coordinated actions of the transcription factors Pax5, IRF4 and Blimp-1, and occurs in a cell division-linked manner (11–14). When B cells acquire increasing levels of Blimp-1 during the differentiation process, they extinguish the B cell programme through downregulation of Pax5 (15–18), arrest cell cycling through repression of c-myc (19) and remodel the sub-cellular organization to support high antibody production through induction of XBP-1 (18, 20). Terminally differentiated PCs require continued expression of IRF4 for survival, as well as Blimp-1, to maintain their ability to produce and secrete antibodies (20).

Alterations in immune responses due to mutations affecting the development or function of immune cells often results in increased susceptibility to infectious diseases. Patients diagnosed with common variable immune deficiency (CVID) typically suffer from recurrent infections, with the respiratory and gastrointestinal tracts commonly affected (21). Diagnosis of CVID is predominantly based on hypogammaglobulinemia, and most patients present in the clinic with a reduced memory B cell pool. This aside, the clinical phenotype of patients diagnosed with primary antibody deficiencies with late B cell defects, such as CVID, is widely heterogeneous and the genetic causes of most cases remain undefined (22–25). However, mutations in the genes encoding Transmembrane Activator and CAML Interactor (TACI), Inducible T cell Costimulator (ICOS) and B cell Activating Factor Receptor (BAFFR) are commonly found, emphasizing the importance of intact B cell activation and signaling (26–28). The recent developments of next-generation sequencing (NGS) methods, such as whole-exome sequencing (WES) and whole-genome sequencing (WGS), have led to the discovery of mutations in many other genes associated with primary immunodeficiencies (25, 29, 30). For example, mutations in several components of the NF-κB and the PI3K signaling pathways, which are activated after BCR signaling, were identified (31–41). Recently, mutations in IRF4 causing complete deficiency or haploinsufficiency have been linked to severe immune defects and predisposition to develop Whipple's disease as a result of infection with *Tropheryma whipplei* bacteria (42, 43). Impaired plasma cell differentiation has been linked to CVID (44), but so far, only a few mutations have been identified as genetic causes for diminished plasma cell generation in PID patients, such as those resulting in IRF4 deficiency and constitutive NF-κB signaling caused by gain-of-function CARD11 mutations (42, 45). Thus, the functional relevance of newly identified mutations and the extent to which they contribute to the clinical phenotype of the patient remains poorly understood.

Here, we established an approach to analyse PC differentiation from primary human B cells in response to *in vitro* stimulation. Firstly, we evaluated different stimulation conditions to determine which was most efficient at inducing PC differentiation. We then developed panels for intracellular staining of transcription factors known to regulate the PC differentiation process (Pax5, IRF4, Blimp-1) and methods to monitor cell proliferation (CellTrace and Ki67) to allow in-depth flow cytometric analyses of the PC differentiation state. We found that, differentiation of human B cells *in vitro* generated IRF4^hi^Pax5^lo^CD38^+^ cells, representing cells committed to PC differentiation. We also found that cryopreservation, which is required for the storage of patient samples, did not markedly affect the ability of the B cells to differentiate into PCs using the stimulation conditions presented here. Thus, this study offers an approach that is applicable to assess B cell function in patient samples for identification of potential plasma cell differentiation defects. We propose that applying such functional assays to CVID cases may allow the stratification of CVID patients into subgroups that do, or do not, display PC differentiation defects, which may help explain how different genetic alterations associate with clinical phenotypes.

## Materials and Equipment

### Medium and Buffers

Ca^2+^- and Mg^2+^-free PBS (Sigma-Aldrich), Ficoll Paque Plus (GE Healthcare), complete medium: RPMI 1640 containing 2mM L-glutamine (HyClone) supplemented with 10% fetal bovine serum (FBS) (HyClone), 0.05 mM β-mercaptoethanol (Gibco Life Technologies), 100 IU penicillin and 100 μg/ml streptomycin (both from Sigma-Aldrich). Red blood cell lysis buffer (RBC) 10X: 1.5M ammonium chloride, 100 mM sodium hydrogen carbonate, 10 mM EDTA, in ultrapure water (in-house), sterile vacuum filtered after preparation using “rapid” Filtermax 150 (TPP). Freezing medium: FBS supplemented with 10% DMSO (Sigma). B cell enrichment buffer: Ca^2+^- and Mg^2+^-free PBS (Sigma-Aldrich) supplemented with 2% FBS and 1 mM EDTA. FACS buffer: Ca^2+^- and Mg^2+^-free PBS (Sigma-Aldrich) supplemented with 2% FBS. ELISA washing buffer: PBS containing 0.05% Tween20 (Sigma-Aldrich). ELISA blocking buffer: PBS containing 2% dry milk (Sigma-Aldrich). ELISA development: TMB substrate (KPL) (Life Technologies) and 1 M H_2_SO_4_ (in-house).

### Reagents

#### B Cell Isolation and Culture

EasySep Human B cell enrichment kit (STEMCELL Technologies), EasySep Human Memory B cell isolation kit (STEMCELL Technologies), Trypan blue stain 0.4% (Invitrogen), unconjugated goat anti-human IgM F(ab')_2_ fragments (Sigma), CpG ODN 2395 (InvivoGen), sCD40L (Peprotech), recombinant human IL-21 (Peprotech).

#### Flow Cytometry

BD Transcription Factor buffer set (BD Biosciences). For antibodies and corresponding dilutions, please see Supplementary Data Sheet 1.

### Equipment

#### Plastics

Sterile serological pipettes (5, 10, 25 ml, Sarstedt), pipette tips (0.2 μl−1,000 μl) (Gilson, Corning), 15 ml and 50 ml Falcon tubes (Corning), 1.5 ml Eppendorf tubes (Sarstedt), flat-bottom 48-well tissue culture plates (Corning), flat-bottom 6-well tissue culture plates (VWR), 5 ml round-bottom polystyrene Falcon tubes (Corning), 0.2 μm single use filter unit (Sartorius), 70 μm cell strainer (VWR), 10 ml syringe (BD Medical), cryotubes (Thermo Scientific), Countess cell counting chamber slides (Invitrogen), flat-bottom 96-well MaxiSorp ELISA plates (Nunc), microplate sealing films and tapes (Fischer Scientific), 50 ml reagent reservoir (VWR).

#### Other

pipet boy, pipettes (0.2–1,000 μl), multipipette, scissors, EasyPlate EasySep magnet (STEMCELL Technologies), ice container, ice, laminar flow hood, benchtop centrifuge, water bath, incubator (37°C 5% CO_2_), cell counter (e.g., Countess II, Invitrogen), flow cytometer (e.g. BD FACSCelesta), microplate washer, spectrophotometer (e.g. Asys Expert 96 ELISA reader, Biochrom Ltd.).

### Software

FlowJo software v9.6.4 (Tree Star), GraphPad Prism v8.

## Methods

### Cell Preparation

Peripheral blood mononuclear cells (PBMCs) were isolated from buffy coats by density gradient centrifugation using Ficoll Paque Plus and washed twice in PBS. Cell suspensions were treated with red blood cell lysis buffer, after which cells were washed twice with PBS, and resuspended in complete medium. Cell suspensions were passed through a 70 μm cell strainer to remove debris. PBMCs were frozen in FBS supplemented with 10% DMSO and stored at −80°C. Frozen PBMCs were thawed at 37°C in a water bath, washed twice in complete medium, and passed through a 70 μm cell strainer. Cell count and viability was performed using Trypan Blue and the automated cell counter Countess II. Total peripheral human B cells were isolated using the EasySep Human B Cell Enrichment Kit (STEMCELL Technologies) according to the manufacturer's protocol. Human memory B cells and naïve B cells were both isolated using the EasySep Human Memory B cell isolation kit (STEMCELL Technologies) according to the manufacturer's protocol. Purity of the total human B cell isolation based on CD20 expression was at least 97% (Supplementary Figures 5A,B).

### *In vitro* B Cell Cultures

B cells were seeded at a density of 2.5 × 10^5^ cells/mL in complete medium in 48-well flat bottom tissue-culture plates for 3.5 and 6 days for flow cytometry. Additionally, supernatants were collected from the day 6 cultures for ELISA. Stimulation I contained 5 μg/mL CpG ODN 2395 (InvivoGen), 1 μg/mL soluble CD40 ligand (sCD40L) (Peprotech), 100 ng/mL recombinant human IL-2 (Peprotech), and 100 ng/mL recombinant human IL-10 (46). Stimulation II contained 5 μg/mL of CpG, 5 μg/mL pokeweed mitogen (PWM) (Sigma-Aldrich), and 1:10.000 protein A from *Staphylococcus aureus* (Sigma-Aldrich) (46). Stimulation III contained 5 μg/mL unconjugated goat anti-human IgM F(ab')_2_ fragments (Sigma), 2,5 μg/mL CpG, 1 μg/mL sCD40L, and 50 ng/mL recombinant human IL-21 (Peprotech) and was adapted from (47). Stimulation IV was adapted from (7) and contained 5 μg/mL unconjugated goat anti-human IgM F(ab')_2_ fragments, 2.5 μg/mL CpG, 50 ng/mL sCD40L, and 5 ng/mL IL-2. At day 4, medium was replaced for stimulation IV with 5 ng/mL IL-2, 10 ng/mL recombinant human IL-4, and 10 ng/mL IL-10 (Peprotech) after washing cells in PBS supplemented with 2% FBS.

### Flow Cytometry

To track cellular divisions, CellTrace Violet (CTV) (Invitrogen) was used at a concentration of 0.25 μM in PBS to label 1 × 10^6^ cells prior to culturing. To block non-specific binding to Fc receptors, cells were incubated with human Fc block reagent (BD). Cells were then stained with different panels of fluorochrome conjugated monoclonal antibodies (Supplementary Table 1) in FACS buffer. For intracellular staining of transcription factors, cells were fixed, permeabilised and stained for 6 h using the transcription factor buffer set (BD) according to the manufacturer's protocol. For intracellular IgG staining, purified IgG was included in the surface panel to block binding to surface IgG. Samples were acquired on a BD FACSCelesta cytometer and data were analyzed by FlowJo software v9.6.4 (Tree Star, Ashland, OR).

### ELISA

ELISA plates (Nunc) were coated with a 1:1,000 dilution of goat anti-human unlabelled IgG (Southern Biotech) in 100 μL PBS per well. After overnight incubation at 4°C, plates were washed six times with washing buffer (PBS+0.05% Tween20) and blocked for 1 h with PBS containing 2% dry milk. Fifty microlitre of culture supernatant was added in a total volume of 150 μL, followed by 3-fold serial dilutions in blocking buffer, and incubated for 2 h at room temperature (RT). Plates were washed six times, and HRP-coupled goat anti-human IgG (Southern Biotech) was added in 100 μL PBS per well in a 1:1,000 dilution, followed by incubation for 1.5 h at RT. After six washes, plates were developed with TMB substrate (KPL) (Life Technologies), and the reaction was stopped with 1 M H_2_SO_4_. The OD was read at 450 nm using an Asys Expert 96 ELISA reader (Biochrom Ltd.).

### Statistics

Differences between groups were analyzed by a Mann-Whitney *U*-test (GraphPad Prism v8). Statistical significance is indicated with ^*^ for *P* ≤ 0.05, ^**^ for *P* ≤ 0.01, ^***^ for *P* ≤ 0.001, and ^****^ for *P*-value ≤ 0.0001.

## Results

### Efficient *in vitro* B Cell Differentiation to PCs by BCR Ligation and Stimulation via TLR9 and CD40

**Figure 1:**
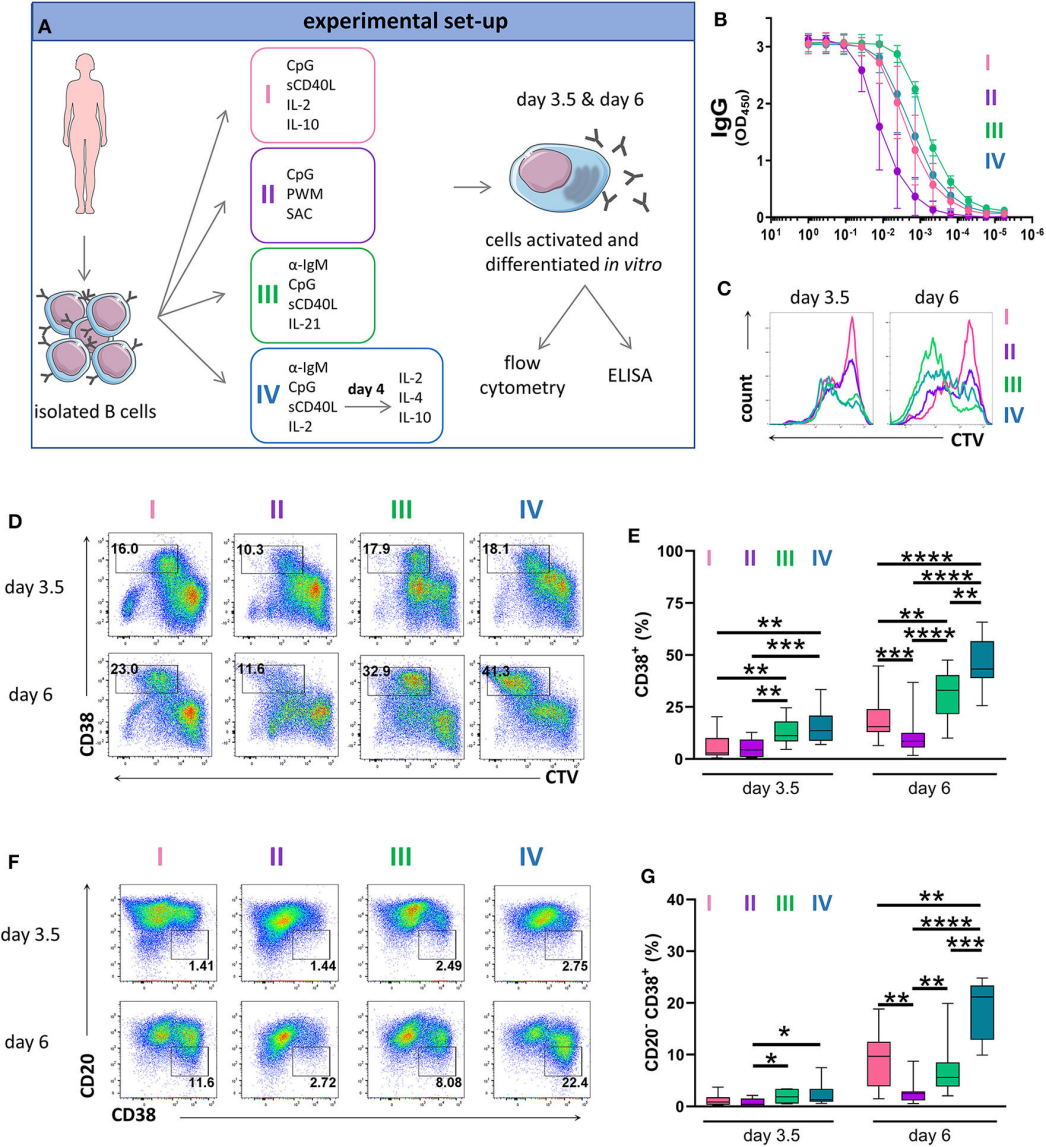
Antibody production and phenotype of primary human B cells after *in vitro* stimulations and culturing. **(A)** Schematic representation of the experimental approach. Total human B cells were isolated from healthy donor blood, stimulated under the indicated conditions and analyzed at day 3.5 and day 6 by flow cytometry to evaluate PC generation. Cells were pre-gated on FSC-A/FSC-W to identify singlets and on FSC-A/SSC-A to identify lymphocytes. The supernatant was collected at day 6 to evaluate IgG production by ELISA. This overview was created using images from Servier Medical Art, which are licensed under a Creative Commons Attribution 3.0 Unported License (http://smart.servier.com). **(B)** Total IgG production from supernatant of cultured cells at day 6 was evaluated by ELISA. Each line and error bar indicate mean ± s.d. **(C)** Cells were labeled with CTV prior to stimulation. Representative histogram plots show an overlay of CTV dilution indicating proliferation in response to the four stimulations. **(D)** Representative plots showing gating strategy for CD38^+^ cell population. Numbers in gates represent cell frequencies. **(E)** Frequencies of CD38^+^ cells are summarized in a box plot with whiskers indicating the minimum and maximum values. Data are pooled from three independent repeats with 5 donors per experiment. Statistical significance was determined by Mann-Whitney *U*-test. **(F)** Representative plots showing gating strategy for CD20^−^ CD38^+^ plasma cell population. Numbers in or adjacent to gates represent cell frequencies. **(G)** Frequencies of CD20^−^ CD38^+^ plasma cell population are summarized in a box plot with whiskers indicating the minimum and maximum values. Data are pooled from three independent repeats with 5 donors per experiment. Statistical significance was determined by Mann-Whitney *U*-test.

To establish an efficient protocol for human PC differentiation, we cultured primary human B cells in four different stimulation cocktails that were adapted from conditions described previously (7, 46, 47). For each condition, we evaluated antibody production by ELISA and PC generation by flow cytometry (Figure 1A). Stimulation I induced TLR9 signaling combined with a stimuli mimicking CD4^+^ T cell help (46). Stimulation II contained a mixture of superantigens known to induce polyclonal B cell activation through non-specific ligation of BCRs and engagement of TLR receptors (46). Stimulation III ligated the BCR and co-stimulatory molecules and provided additional cytokine stimulation via IL-21, which is an important inducer of Blimp-1 expression, particularly in combination with CD40 ligation (47). Stimulation IV utilized a 2-step approach where, at day 4, the activation medium was replaced with medium supplemented with IL-2, IL-4, and IL-10 to assist cells at the terminal stage of differentiation (7). The optimal concentration of IL-2 for enhancing PC differentiation was determined after titration (Supplementary Figure 1). When assessing IgG immunoglobulin secretion after 6 days of culture we found that the highest IgG levels were achieved by stimulation III (Figure 1B).

To assess proliferation, we labeled B cells with CellTrace Violet (CTV) prior to culturing. Proliferation was observed in all cultures at day 3.5 but was most notable in the cultures treated with stimulation III at day 6 (Figure 1C and Supplementary Figure 2). Cells that upregulated CD38 whilst proliferating were described to represent a population of (pre)plasma blasts and plasma cells (13, 48). At day 3.5, stimulation III and IV generated similar frequencies of CD38^+^ cells with lower frequencies observed for stimulation I and II. At day 6, we observed the highest frequency of CD38^+^ cells for stimulation IV (Figures 1D,E). During differentiation, B cells downregulate surface CD20 expression on CD38^+^ cells (48). Again, at day 3.5 we observed no differences between stimulation III and IV while at day 6, most CD20^−^ CD38^+^ cells were obtained for stimulation IV (Figures 1F,G). Thus, stimulation III and IV were most efficient at generating CD38^+^ cells and were both suitable for assessing PC differentiation *in vitro*, with stimulation III resulting in higher levels of IgG secretion.

### *In vitro* Stimulation of Human B Cells Generated Populations With Distinct IRF4 and Pax5 Expression Levels

**Figure 2:**
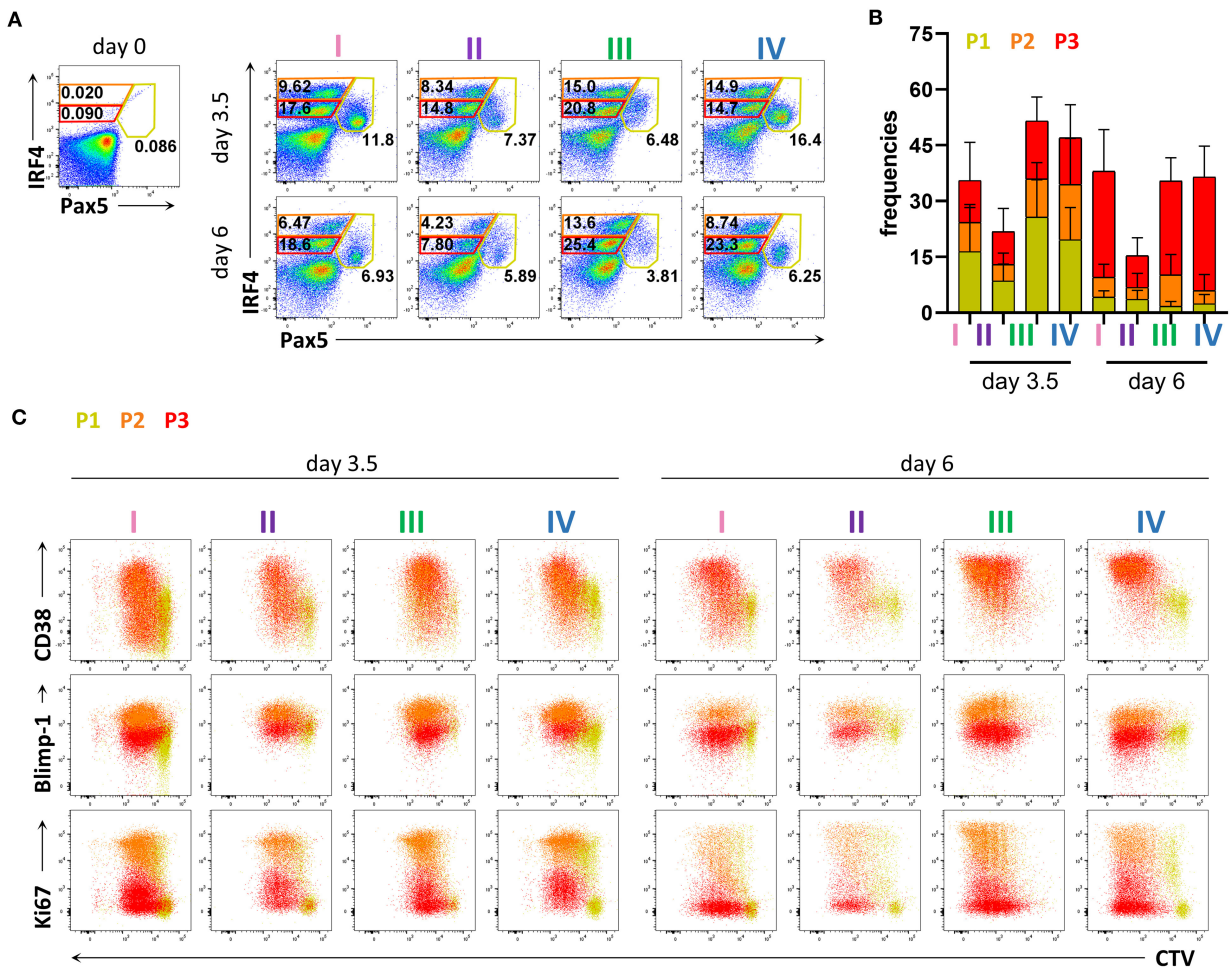
Flow cytometric analysis of transcription factor expression in human primary B cells after *in vitro* differentiation. Total human B cells from healthy donors were stimulated under conditions described in Figure 1A and analyzed by flow cytometry at days 3.5 and 6. Cells were pre-gated on FSC-A/FSC-W to identify singlets and on FSC-A/SSC-A to identify lymphocytes. **(A)** Representative plots showing gating strategy for IRF4^lo^ Pax5^hi^ (P1), IRF4^hi^ Pax5^lo^ (P2), and IRF4^int^ Pax5^lo^ (P3) populations. Numbers in or adjacent to gates indicate cell frequencies. **(B)** Frequencies of IRF4^low^ Pax5^hi^ (P1), IRF4^hi^ Pax5^lo^ (P2), and IRF4^int^ Pax5^lo^ (P3) cells. Bars and error bars indicate mean ± s.d. Data are pooled from three independent repeats with 5 donors per experiment. **(C)** Representative plot overlays showing CD38, Blimp-1 and Ki67 expression for the IRF4^low^ Pax5^hi^ (P1), IRF4^hi^ Pax5^lo^ (P2), and IRF4^int^ Pax5^lo^ (P3) populations.

To evaluate PC differentiation *in vitro* at the transcription factor level, we next assessed the expression of IRF4 and Pax5. We identified three populations present at both 3.5 and 6 days of stimulation; IRF4^lo^Pax5^hi^, IRF4^hi^Pax5^lo^, and IRF4^int^Pax5^lo^ referred to hereafter as P1, P2, and P3, respectively (Figure 2A). Between day 3.5 and day 6, we observed a decrease in the frequency of population P1, and an increase in the frequency of population P3 (Figures 2A,B). Both populations P2 and P3 contained CD27^+^CD38^+^ cells suggesting that these two populations represent plasma blasts and plasma cells (Figure 2C and Supplementary Figure 3A). In support of this finding, we detected intracellular IgG in 28.58 ± 11.65% of P2 cells and 29.75 ± 10.73% of P3 cells at day 3.5, which increased to *ca*. 40% for both populations at day 6 (Supplementary Figure 3B). Interestingly, at day 6, the majority of P3 cells exhibited lower expression of Blimp-1 and Ki67, indicating that these cells were no longer actively cycling. In contrast, population P2 had high expression of both Blimp-1 and Ki67 and thus resembled plasma cells phenotypically and transcriptionally (Figure 2C and Supplementary Figure 4). These results indicate that human plasma cell differentiation *in vitro* can be characterized by three stages defined by Pax5 upregulation (P1), IRF4 upregulation and Pax5 repression (P2), and finally, decrease in IRF4 expression (P3). Of these three populations, P2 exhibits a phenotype most characteristic of plasma cells as evidenced by high expression of both IRF4 and Blimp-1.

### Naïve and Memory B Cells Exhibited Distinct Differentiation Kinetics

**Figure 3:**
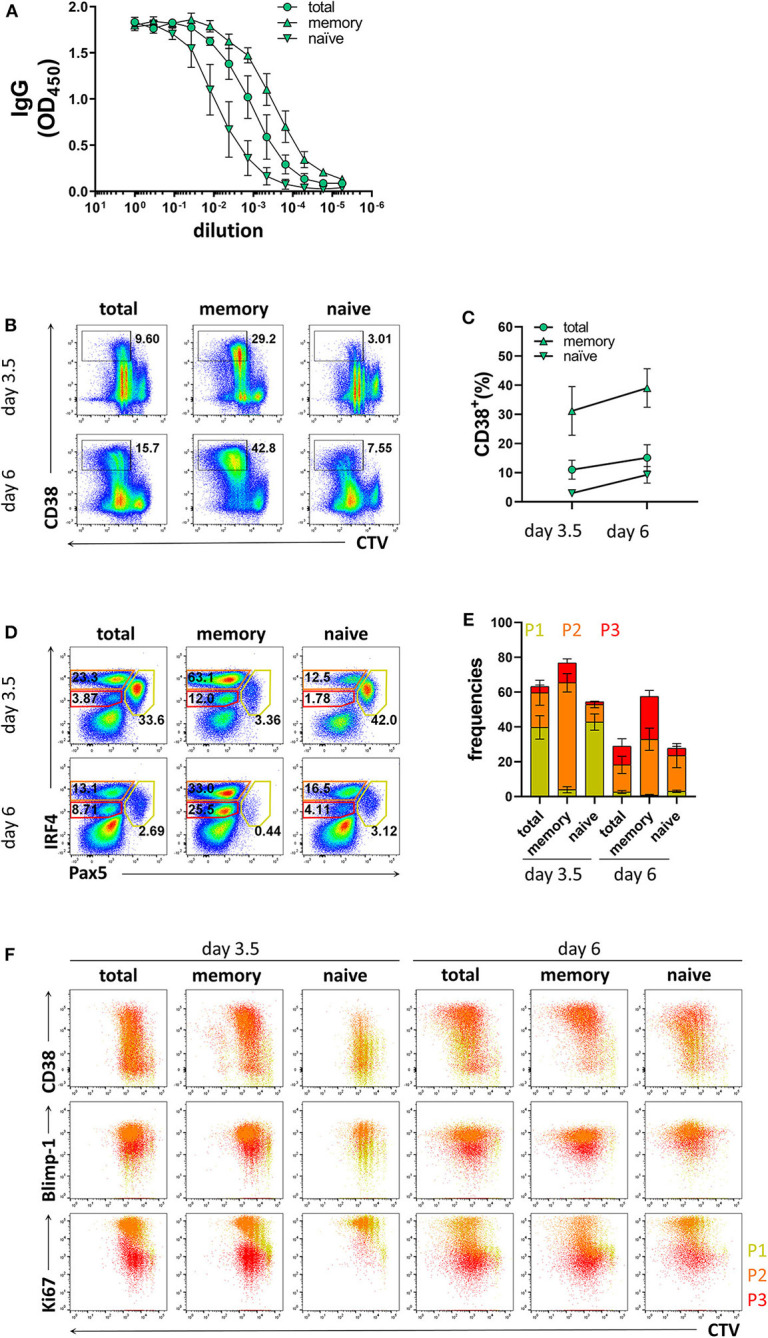
Differentiation of memory B cells and naïve B cells *in vitro*. Memory B cells, naïve B cells, and total B cells were isolated from healthy donors, cultured with stimulation III, and analyzed by flow cytometry at day 3.5 and day 6. Cells were pre-gated on FSC-A/FSC-W to identify singlets and on FSC-A/SSC-A to identify lymphocytes. **(A)** Total IgG production from supernatant of cultured cells at day 6 was evaluated by ELISA. Each line and error bar indicate mean ± s.d. **(B)** Representative plots showing gating strategy for CD38^+^ population. Numbers adjacent to gates indicate cell frequencies. **(C)** Frequencies of CD38^+^ cells with symbols and error bars indicating mean ± s.d. **(D)** Representative plots showing gating strategy for IRF4^lo^ Pax5^hi^ (P1), IRF4^hi^ Pax5^lo^ (P2), and IRF4^int^ Pax5^lo^ (P3) populations. Numbers in or adjacent to gates represent cell frequencies. **(E)** Frequencies of IRF4^low^ Pax5^hi^ (P1), IRF4^hi^ Pax5^lo^ (P2), and IRF4^int^ Pax5^lo^ (P3) populations. Bars and error bars indicate mean ± s.d. **(F)** Plot overlays showing CD38, Blimp-1 and Ki67 expression for IRF4^low^ Pax5^hi^ (P1), IRF4^hi^ Pax5^lo^ (P2), and IRF4^int^ Pax5^lo^ (P3) populations. Data are representative of three independent repeats with 3–5 donors per experiment.

As memory B cells have the ability to respond to cognate antigen and differentiate into PCs with faster kinetics compared to naïve B cells (9, 10, 49), the ratio between memory and naïve B cells in the starting PBMC sample may affect the efficiency of PC differentiation in stimulated B cell cultures. Since the frequencies of memory B cells may vary between donors and, particularly for CVID patients, may be lower than those observed in healthy donors, we compared the differentiation kinetics of cultures enriched for memory B cells or naïve B cells. Based on CD27 expression, purity of the enriched populations was 91.7 ± 6.5% and 96.2 ± 1.4% for memory B cells and naïve B cells, respectively (Supplementary Figures 5A,B). The B cell enrichment methods could potentially result in the inclusion of plasma cells. However, we found very few CD20^−^CD38^+^ cells and no distinguishable P1, P2, and P3 populations in enriched memory and naïve B cells (Supplementary Figures 5C,D). As expected, memory B cells were most efficient at producing IgG and generating CD38^+^ cells (Figures 3A–C). In addition, they generated higher frequencies of the P2 population already at day 3.5 (Figures 3D,E). Even though naïve B cells generated lower frequencies of CD38^+^ cells and populations P2 and P3 compared to total B cells at day 3.5, at day 6 the frequencies of CD38^+^ cells and population P2 were similar to total B cell cultures (Figures 3B,F). Taken together, these results demonstrate that the differentiation conditions used here induced PC differentiation from both memory and naïve B cells and are thus applicable to patient samples with low memory B cell frequencies.

### The Efficiency of *in vitro* B Cell Differentiation to PCs Was Retained in Cryopreserved PBMCs

**Figure 4:**
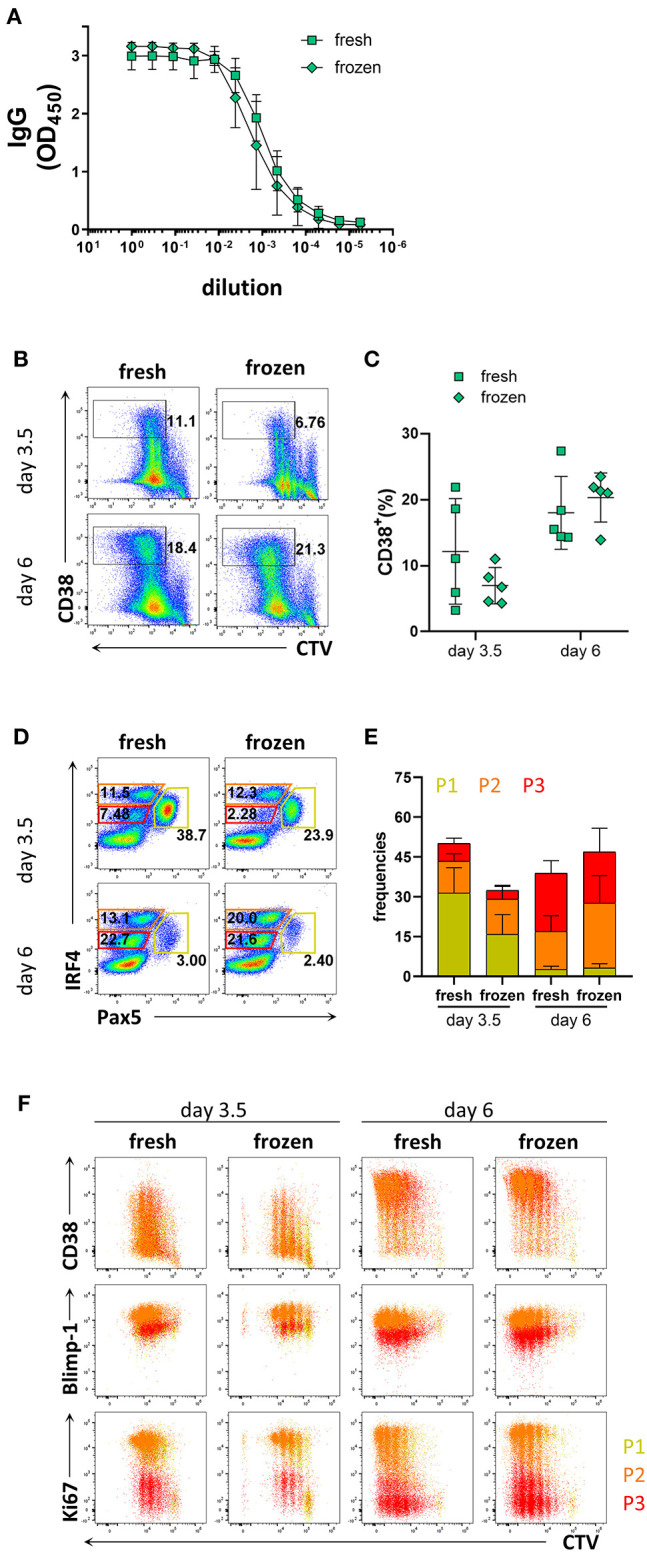
Comparison of PC differentiation of B cells isolated freshly from PBMCs or from cryopreserved PBMCs. Total human B cells were isolated from fresh PBMCs and cryopreserved PBMCs. IgG production and PC differentiation were assessed in response to stimulation III. Cells were pre-gated on FSC-A/FSC-W to identify singlets and on FSC-A/SSC-A to identify lymphocytes. **(A)** Total IgG production from supernatant of cultured cells at day 6 was evaluated by ELISA. Each line and error bar indicate mean ± s.d. **(B)** Representative plots showing gating strategy for CD38^+^ population. Numbers adjacent to gates indicate cell frequencies. **(C)** Frequencies of CD38^+^ cells summarized with bars and error bars indicating mean ± s.d. **(D)** Representative plots showing gating strategy for IRF4^lo^ Pax5^hi^ (P1), IRF4^hi^ Pax5^lo^ (P2), and IRF4^int^ Pax5^lo^ (P3) populations. Numbers in or adjacent to gates represent cell frequencies. **(E)** Frequencies of IRF4^low^ Pax5^hi^ (P1), IRF4^hi^ Pax5^lo^ (P2), and IRF4^int^ Pax5^lo^ (P3) populations. Bars and error bars indicate mean ± s.d. **(F)** Plot overlays showing CD38, Blimp-1 and Ki67 expression for IRF4^low^ Pax5^hi^ (P1), IRF4^hi^ Pax5^lo^ (P2), and IRF4^int^ Pax5^lo^ (P3) populations. Data are representative of three independent repeats with 4–5 donors per experiment.

As collection of patient materials often involves freezing samples, we assessed whether the process of freezing and thawing affected the differentiation potential of B cells into PCs. To this end, B cells isolated from freshly obtained donor blood were compared to B cells isolated from frozen PBMCs. B cell purity based on CD20 expression was 98.6 ± 1.5% from fresh samples, and 98.4 ± 1.7% from frozen samples (Supplementary Figures 6A,B). The prevalence of naïve and memory B cells obtained from fresh and frozen PBMC samples after B cell isolation was similar; 65.1 ± 9.3% from fresh samples and 62.5 ± 12.6% from frozen samples for naïve B cells, and 24.7 ± 8.3% from fresh samples and 21.3 ± 5.9% from frozen samples for memory B cells (Supplementary Figures 6C,D). After 6 days of culture with stimulation III, similar levels of IgG were detected from the supernatant from frozen and fresh samples (Figure 4A). B cells isolated from frozen PBMCs proliferated to a similar extent as those from fresh PBMCs but exhibited a tendency to generate less CD38^+^ cells (Figures 4B,C). Frequencies of populations P1, P2, and P3 and their phenotypes were also similar between fresh and frozen samples (Figures 4D–F). Hence, cryopreservation of PBMC samples did not appear to affect the ability of the B cells to differentiate into PCs and produce antibodies after *in vitro* culturing using the stimulation III conditions described here.

## Discussion

**Figure 5:**
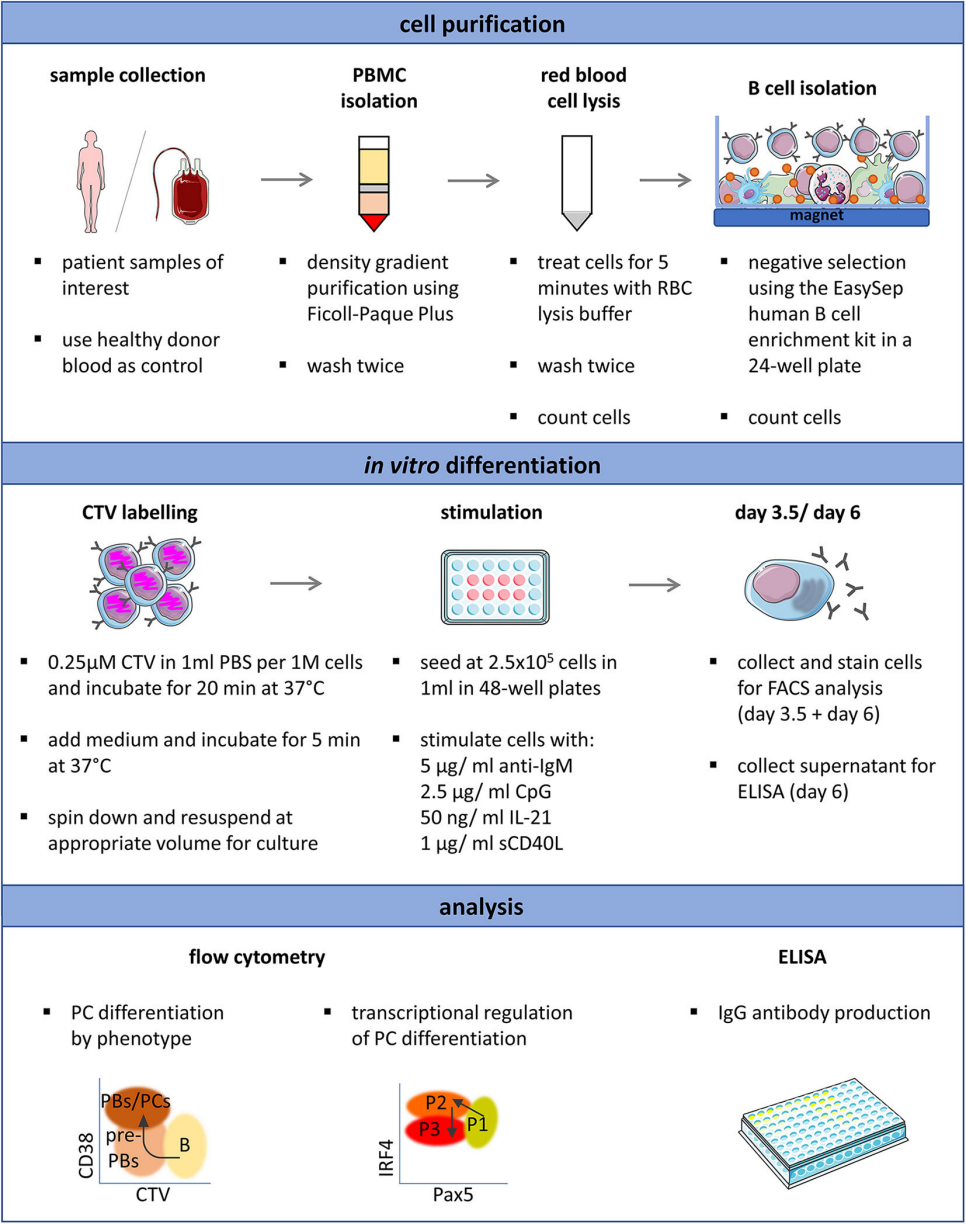
Schematic representation of the optimized protocol for the *in vitro* induction and analysis of plasma cell differentiation from human primary B cells. Proliferation (CTV), CD38, CD20, Pax5, IRF4, Blimp-1, and Ki67 expression can be analyzed simultaneously and requires 2.5 × 10^5^ isolated B cells per time point. This overview was created using images from Servier Medical Art, which are licensed under a Creative Commons Attribution 3.0 Unported License (http://smart.servier.com).

CVID comprises a family of primary immunodeficiencies with patients suffering from increased susceptibility to infections due to hypogammaglobulinemia and impaired antibody responses (21). Most underlying genetic causes have been described in the receptors and ligands required for B cell signaling or B-T cell interactions, thus interfering with B cell activation and differentiation (28). The association between mutations in certain genes and the development of CVID is only well established for 2–10% of cases (21). Whole genome or exome sequencing efforts have resulted in the discovery of novel mutations in genes that are associated with CVID (29, 30). However, how most of these mutations impact B cell differentiation and function remains poorly understood. Immunophenotypic characterization of immunodeficient patients is useful for defining subcategories as patients can present with variable B cell numbers, and reduced frequencies of isotype switched memory B cells and plasma cells (50, 51). However, immunophenotyping does not address potential causes of B cell abnormalities. These studies include analysis of transcription factors involved in PC differentiation and B cell proliferation. Based on these parameters, and in contrast to standard analysis of PCs (CD38^+^CD27^−^IgM^−^), our assay may be able to further differentiate CVID patients into subgroups displaying more specific defects. In this study, we established flow cytometry-based protocols for the analysis of human plasma cell differentiation of primary B cells, which are suitable for the analysis of CVID patient samples (Figure 5). Our results showed that *in vitro* PC differentiation was most efficiently induced by combining BCR and TLR9 ligation with stimuli mimicking T cell help, which induced the production of IRF4^hi^Pax5^lo^CD38^+^ cells, a phenotype typical of B cells that have committed to PC differentiation. The next best stimulation conditions based on our evaluation was a two-step approach (stimulation IV), which also efficiently generated PC differentiation. However, this protocol was less practical, and the washing and reseeding steps resulted in a lower yield of secreted antibodies due to the need to change medium on day 4.

Since the frequency of memory B cells present in some CVID patients may be lower than that observed in healthy donors, we considered it important to investigate the relative contribution of naïve vs. memory B cells to plasma cell differentiation under the conditions used. In the present study, the frequency of memory B cells was 24.7 ± 8.3% and the frequency of naïve B cells was 65.1 ± 9.3% based on expression of CD27 in the different donors (Supplementary Figures 5A,B). Despite this variation, plasma cell differentiation in naïve B cell cultures was readily detectable as CD38^+^IRF4^hi^Pax5^lo^ cells were generated, albeit at a lower pace compared to total B cell cultures, suggesting that the method described is applicable also to donors with low memory B cell frequencies. In addition, B cells from cryopreserved PBMCs retained the ability to generate CD38^+^IRF4^hi^Pax5^lo^ cells, though at day 3.5 we observed a tendency toward lower frequencies of CD38^+^ cells and IRF4^int^Pax5^lo^ cells compared to those from fresh samples.

IRF4 is required for initiating plasma blast differentiation, whereas Blimp-1 is critical for converting the gene expression state of the cell to being PC-specific. Blimp-1 is absolutely indispensable for antibody production and secretion through its regulation of both the unfolded protein response (UPR) and cellular metabolism (18, 20). During differentiation, the B cell-specific gene program is extinguished, which is reflected by decreased Pax5 levels (15, 16). Absence or reduced frequencies of CD38^+^ cells, altered or reduced expression of IRF4 or Blimp-1, or failed downregulation of Pax5 after *in vitro* stimulation compared to healthy donor controls could help identify defects in plasma cell differentiation in patients. Additionally, accelerated or premature upregulation of Blimp-1 could indicate impaired survival of differentiating cells committed to PC fate (52). Identifying and characterizing mutations in genes involved in PC differentiation might help explain alterations in memory and PC frequencies, hypogammaglobulinemia, and the increased susceptibility of infections observed in CVID patients. In summary, establishing and applying functional PC differentiation assays to patient samples may be used to stratify patients suffering from primary antibody deficiencies with late B cell defects into subgroups to increase our understanding of disease phenotypes.

## Data Availability Statement

The raw data supporting the conclusions of this article will be made available by the authors, without undue reservation.

## Ethics Statement

Ethical review and approval was not required for the study on human participants in accordance with the local legislation and institutional requirements. Written informed consent for participation was not required for this study in accordance with the national legislation and the institutional requirements.

## Author Contributions

SK and GKH conceived and directed the study. SK performed experiments, data collection, analysis and interpretation with help of MÁ. All authors discussed the results. SK wrote the manuscript with critical feedback from MÁ, GP, and GKH.

## Conflict of Interest

The authors declare that the research was conducted in the absence of any commercial or financial relationships that could be construed as a potential conflict of interest.

## Supplementary Material

Supplementary Figure 1 Titration of IL-2. Human primary B cells were cultured with stimulation IV containing 5, 10, 25, 50, or 100 ng IL-2 with the same concentration used in the media for day 0–4 as well as day 4–6. Cells were pre-gated on FSC-A/FSC-W to identify singlets and on FSC-A/SSC-A to identify lymphocytes. **(A)** Representative flow cytometry plots showing gating strategy for CD38^+^ cells. Numbers adjacent to gates indicate cell frequencies. **(B)** Frequencies of CD38^+^ cells are summarized in a box plot with whiskers indicating the minimum and maximum values. Statistical significance was determined using Mann-Whitney *U*-test. Data are representative of two experiments with 5 donors.

Supplementary Figure 2 Cellular divisions of stimulated B cells. Total B cells were isolated from healthy donor blood, labeled with CTV, stimulated under conditions described in Figure 1A, and analyzed at day 3.5 and day 6 by flow cytometry. Cells were pre-gated on FSC-A/FSC-W to identify singlets and on FSC-A/SSC-A to identify lymphocytes. Representative histogram overlays show cellular divisions from five donors based on CTV dilution. Data are representative of three independent repeats with 5 donors per experiment.

Supplementary Figure 3 Population P2 and P3 contain cells with a plasma blast/plasma cell phenotype. Total B cells were isolated from healthy donor blood, cultured with stimulation III, and analyzed at day 3.5 and day 6 by flow cytometry. Cells were pre-gated on FSC-A/FSC-W to identify singlets, on FSC-A/SSC-A to identify lymphocytes, on Live/Dead Green^−^ to exclude dead cells, and on IRF4 and Pax5 expression to identify populations P1, P2, and P3. **(A)** Representative plots showing gating strategy for CD27^+^ CD38^+^ cells (left). Numbers adjacent to gates indicate cell frequencies. Frequencies of CD27^+^ CD38^+^ cells are summarized in a box plot with whiskers indicating the minimum and maximum values (right). **(B)** Representative plots showing gating strategy for intracellular IgG^+^ cells (left). Numbers adjacent to gates indicate cell frequencies. Frequencies of IgG^+^ cells are summarized in a box plot with whiskers indicating the minimum and maximum values (right). Data are pooled from two independent repeats with 3 donors per experiment.

Supplementary Figure 4 Flow cytometric analysis of CD38, Blimp-1 and Ki67 expression in the P1, P2, and P3 populations. Total human B cells from healthy donors were stimulated under conditions described in Figure 1A and analyzed by flow cytometry at days 3.5 and 6. Cells were pre-gated on FSC-A/FSC-W to identify singlets, on FSC-A/SSC-A to identify lymphocytes, and on IRF4/ Pax5 expression to identify the P1, P2, and P3 populations. Representative plots showing CD38, Blimp-1 and Ki67 expression for the IRF4^low^ Pax5^hi^ (P1), IRF4^hi^ Pax5^lo^ (P2), and IRF4^int^ Pax5^lo^ (P3) populations.

Supplementary Figure 5 Purity of naïve and memory B cell populations before and after enrichment. **(A)** Representative plots showing gating strategy for CD20^+^ CD38^+^ CD27^−^ naïve and CD20^+^ CD38^+^ CD27^+^ memory B cell populations from fresh PBMCs and enriched populations. Cells were pre-gated on FSC-A/FSC-W to identify singlets and on FSC-A/SSC-A to identify lymphocytes. Numbers adjacent to gates indicate cell frequencies. **(B)** Frequencies of CD20^+^ CD38^+^ CD27^−^ naïve and CD20^+^ CD38^+^ CD27^+^ memory B cell populations before and after enrichment. Data are representative of three independent repeats with 4-5 donors per experiment. **(C)** Representative plots showing gating strategy for CD20^−^CD38^+^ cells and populations P1, P2, and P3 based on IRF4 and Pax5 expression from total, memory, and naïve B cells immediately after enrichment. Cells cultured for 3.5 days with stimulation III are included as gating control. Cells were pre-gated on FSC-A/FSC-W to identify singlets, on FSC-A/SSC-A to identify lymphocytes, and on Live/Dead Green^−^ to exclude dead cells. Numbers adjacent to gates indicate cell frequencies. **(D)** Frequencies of CD20^−^CD38^+^ cells. Data are representative of two independent repeats with 3 donors per experiment.

Supplementary Figure 6 Naïve and memory B cell populations in pre- and post-isolation samples from fresh PBMCs and frozen PBMCs. Cells were pre-gated on FSC-A/FSC-W to identify singlets and on FSC-A/SSC-A to identify lymphocytes. **(A)** Representative histogram showing gating strategy for the CD20^+^ population before and after isolation. **(B)** Frequencies of CD20^+^ B cells before and after isolation are shown for four samples from fresh PBMCs and four samples from frozen PBMCs. **(C)** Representative plots showing gating strategy for CD20^+^ CD38^+^ CD27^−^ naïve and CD20^+^ CD38^+^ CD27^+^ memory B cell populations from fresh PBMCs (left panel) and frozen PBMCs (right panel). Numbers adjacent to gates represent the frequencies of cells gated on. **(D)** Graph showing frequencies of CD20^+^ CD38^+^ CD27^−^ naïve and CD20^+^ CD38^+^ CD27^+^ memory B cell populations before and after isolation. Data are representative of three independent repeats with 4–5 donors per experiment.

## References

[1] MacLennan IC. Germinal centers. Annu Rev Immunol. (1994) 12:117–39. 10.1146/annurev.iy.12.040194.001001 8011279

[2] Vinuesa CG Tangye SG Moser B Mackay CR. Follicular B helper T cells in antibody responses and autoimmunity. Nat Rev Immunol. (2005) 5:853–65. 10.1038/nri1714 16261173

[3] Rousset F Garcia E Defrance T Péronne C Vezzio N Hsu D. IL10 is a potent growth and differentiation factor for activated human B lymphocytes. Proc Natl Acad Sci USA. (1992) 89:3. 10.1073/pnas.89.5.1890 1371884 PMC48559

[4] Ettinger R Sims GP Fairhurst AM Robbins R da Silva YS Spolski R. IL-21 induces differentiation of human naive and memory B cells into antibody-secreting plasma cells. J Immunol. (2005) 175:7867–79. 10.4049/jimmunol.175.12.7867 16339522

[5] Diehl SA Schmidlin H Nagasawa M van Haren SD Kwakkenbos MJ Yasuda E. STAT3-mediated up-regulation of BLIMP1 Is coordinated with BCL6 down-regulation to control human plasma cell differentiation. J Immunol. (2008) 180:4805–15. 10.4049/jimmunol.180.7.4805 18354204 PMC2396731

[6] Avery DT Deenick EK Ma CS Suryani S Simpson N Chew GY. B cell-intrinsic signaling through IL-21 receptor and STAT3 is required for establishing long-lived antibody responses in humans. J Exp Med. (2010) 207:155–71. 10.1084/jem.20091706 20048285 PMC2812540

[7] Le Gallou S Caron G Delaloy C Rossille D Tarte K Fest T. IL-2 requirement for human plasma cell generation: coupling differentiation and proliferation by enhancing MAPK-ERK signaling. J Immunol. (2012) 189:161–73. 10.4049/jimmunol.1200301 22634617

[8] Ding BB Bi E Chen H Yu JJ Ye BH. IL-21 and CD40L synergistically promote plasma cell differentiation through upregulation of blimp-1 in human B cells. J Immunol. (2013) 190:1827–36. 10.4049/jimmunol.1201678 23325890 PMC3563840

[9] Good KL Tangye SG. Decreased expression of Kruppel-like factors in memory B cells induces the rapid response typical of secondary antibody responses. Proc Natl Acad Sci USA. (2007) 104:13420–5. 10.1073/pnas.0703872104 17673551 PMC1948953

[10] Kometani K Nakagawa R Shinnakasu R Kaji T Rybouchkin A Moriyama S. Repression of the transcription factor Bach2 contributes to predisposition of IgG1 memory B cells toward plasma cell differentiation. Immunity. (2013) 39:136–47. 10.1016/j.immuni.2013.06.011 23850379

[11] Hodgkin PD Lee JH Lyons AB. B cell differentiation and isotype switching is related to division cycle number. J Exp Med. (1996) 184:277–81. 10.1084/jem.184.1.277 8691143 PMC2192686

[12] Tangye SG Avery DT Hodgkin PD. A division-linked mechanism for the rapid generation of Ig-secreting cells from human memory B cells. J Immunol. (2003) 170:261–9. 10.4049/jimmunol.170.1.261 12496408

[13] Caron G Hussein M Kulis M Delaloy C Chatonnet F Pignarre A. Cell-cycle-dependent reconfiguration of the DNA methylome during terminal differentiation of human B cells into plasma cells. Cell Rep. (2015) 13:1059–71. 10.1016/j.celrep.2015.09.051 26565917

[14] Lin WH Adams WC Nish SA Chen YH Yen B Rothman NJ. Asymmetric PI3K signaling driving developmental and regenerative cell fate bifurcation. Cell Rep. (2015) 13:2203–18. 10.1016/j.celrep.2015.10.072 26628372 PMC4685001

[15] Lin KI Angelin-Duclos C Kuo TC Calame K. Blimp-1-dependent repression of Pax-5 is required for differentiation of B cells to immunoglobulin M-secreting plasma cells. Mol Cell Biol. (2002) 22:4771–80. 10.1128/MCB.22.13.4771-4780.2002 12052884 PMC133916

[16] Shaffer AL Lin KI Kuo TC Yu X Hurt EM Rosenwald A. Blimp-1 orchestrates plasma cell differentiation by extinguishing the mature B cell gene expression program. Immunity. (2002) 17:51–62. 10.1016/S1074-7613(02)00335-7 12150891

[17] Delogu A Schebesta A Sun Q Aschenbrenner K Perlot T Busslinger M. Gene repression by Pax5 in B cells is essential for blood cell homeostasis and is reversed in plasma cells. Immunity. (2006) 24:269–81. 10.1016/j.immuni.2006.01.012 16546096

[18] Minnich M Tagoh H Bonelt P Axelsson E Fischer M Cebolla B. Multifunctional role of the transcription factor Blimp-1 in coordinating plasma cell differentiation. Nat Immunol. (2016) 17:331–43. 10.1038/ni.3349 26779602 PMC5790184

[19] Lin Y Wong K Calame K. Repression of c-myc transcription by Blimp-1, an inducer of terminal B cell differentiation. Science. (1997) 276:596–9. 10.1126/science.276.5312.596 9110979

[20] Tellier J Shi W Minnich M Liao Y Crawford S Smyth GK. Blimp-1 controls plasma cell function through the regulation of immunoglobulin secretion and the unfolded protein response. Nat Immunol. (2016) 17:323–30. 10.1038/ni.3348 26779600 PMC4757736

[21] Bonilla FA Barlan I Chapel H Costa-Carvalho BT Cunningham-Rundles C de la Morena MT. International consensus document (ICON): common variable immunodeficiency disorders. J Allergy Clin Immunol Pract. (2016) 4:38–59. 10.1016/j.jaip.2015.07.025 26563668 PMC4869529

[22] Cunningham-Rundles C. The many faces of common variable immunodeficiency. Hematology Am Soc Hematol Educ Program. (2012) 2012:301–5. 10.1182/asheducation.V2012.1.301.3798316 23233596 PMC4066657

[23] Durandy A Kracker S Fischer A. Primary antibody deficiencies. Nat Rev Immunol. (2013) 13:519–33. 10.1038/nri3466 23765059

[24] Jolles S. The variable in common variable immunodeficiency: a disease of complex phenotypes. J Allergy Clin Immunol Pract. (2013) 1:545–56; quiz 557. 10.1016/j.jaip.2013.09.015 24565700

[25] Thaventhiran JED Lango Allen H Burren OS Rae W Greene D Staples E. Whole-genome sequencing of a sporadic primary immunodeficiency cohort. Nature. (2020) 583:90–5. 10.1038/s41586-020-2265-1 32499645 PMC7334047

[26] Castigli E Wilson SA Garibyan L Rachid R Bonilla F Schneider L. TACI is mutant in common variable immunodeficiency and IgA deficiency. Nat Genet. (2005) 37:829–34. 10.1038/ng1601 16007086

[27] Salzer U Chapel HM Webster AD Pan-Hammarstrom Q Schmitt-Graeff A Schlesier M. Mutations in TNFRSF13B encoding TACI are associated with common variable immunodeficiency in humans. Nat Genet. (2005) 37:820–8. 10.1038/ng1600 16007087

[28] Bogaert DJ Dullaers M Lambrecht BN Vermaelen KY De Baere E Haerynck F. Genes associated with common variable immunodeficiency: one diagnosis to rule them all? J Med Genet. (2016) 53:575–90. 10.1136/jmedgenet-2015-103690 27250108

[29] Maffucci P Filion CA Boisson B Itan Y Shang L Casanova JL. Genetic diagnosis using whole exome sequencing in common variable immunodeficiency. Front Immunol. (2016) 7:220. 10.3389/fimmu.2016.00220 27379089 PMC4903998

[30] Meyts I Bosch B Bolze A Boisson B Itan Y Belkadi A. Exome and genome sequencing for inborn errors of immunity. J Allergy Clin Immunol. (2016) 138:957–69. 10.1016/j.jaci.2016.08.003 27720020 PMC5074686

[31] Conley ME Dobbs AK Quintana AM Bosompem A Wang YD Coustan-Smith E. Agammaglobulinemia and absent B lineage cells in a patient lacking the p85alpha subunit of PI3K. J Exp Med. (2012) 209:463–70. 10.1084/jem.20112533 22351933 PMC3302225

[32] Chen K Coonrod EM Kumanovics A Franks ZF Durtschi JD Margraf RL. Germline mutations in NFKB2 implicate the noncanonical NF-kappaB pathway in the pathogenesis of common variable immunodeficiency. Am J Hum Genet. (2013) 93:812–24. 10.1016/j.ajhg.2013.09.009 24140114 PMC3824125

[33] Lucas CL Zhang Y Venida A Wang Y Hughes J McElwee J. Heterozygous splice mutation in PIK3R1 causes human immunodeficiency with lymphoproliferation due to dominant activation of PI3K. J Exp Med. (2014) 211:2537–47. 10.1084/jem.20141759 25488983 PMC4267241

[34] Fliegauf ML Bryant V Frede N Slade C Woon ST. Haploinsufficiency of the NF-κB1 Subunit p50 in common variable immunodeficiency. Am J Human Genet. (2015) 97:389–403. 10.1016/j.ajhg.2015.07.008 26279205 PMC4564940

[35] Boztug H Hirschmugl T Holter W Lakatos K Kager L Trapin D. NF-kappaB1 haploinsufficiency causing immunodeficiency and EBV-driven lymphoproliferation. J Clin Immunol. (2016) 36:533–40. 10.1007/s10875-016-0306-1 27338827 PMC4940442

[36] Kuehn HS Niemela JE Sreedhara K Stoddard JL Grossman J Wysocki CA. Novel nonsense gain-of-function NFKB2 mutations associated with a combined immunodeficiency phenotype. Blood. (2017) 130:1553–64. 10.1182/blood-2017-05-782177 28778864 PMC5620416

[37] Sharfe N Karanxha A Dadi H Merico D Chitayat D Herbrick JA. Dual loss of p110delta PI3-kinase and SKAP. (KNSTRN) expression leads to combined immunodeficiency and multisystem syndromic features. J Allergy Clin Immunol. (2018) 142:618–29. 10.1016/j.jaci.2017.10.033 29180244

[38] Sogkas G Fedchenko M Dhingra A Jablonka A Schmidt RE Atschekzei F. Primary immunodeficiency disorder caused by phosphoinositide 3-kinase delta deficiency. J Allergy Clin Immunol. (2018) 142:1650–1653.e1652. 10.1016/j.jaci.2018.06.039 30040974

[39] Tang P Upton JEM Barton-Forbes MA Salvadori MI Clynick MP Price AK. Autosomal recessive agammaglobulinemia due to a homozygous mutation in PIK3R1. J Clin Immunol. (2018) 38:88–95. 10.1007/s10875-017-0462-y 29178053

[40] Cohen SB Bainter W Johnson JL Lin TY Wong JCY Wallace JG. Human primary immunodeficiency caused by expression of a kinase-dead p110delta mutant. J Allergy Clin Immunol. (2019) 143:797–799.e792. 10.1016/j.jaci.2018.10.005 30336224 PMC6387453

[41] Klemann C Camacho-Ordonez N Yang L Eskandarian Z Rojas-Restrepo JL Frede N. Clinical and immunological phenotype of patients with primary immunodeficiency due to damaging mutations in NFKB2. Front Immunol. (2019) 10:297. 10.3389/fimmu.2019.00297 30941118 PMC6435015

[42] Bravo García-Morato M Aracil Santos FJ Briones AC Blázquez Moreno A Del Pozo Maté Á Domínguez-Soto Á. New human combined immunodeficiency caused by interferon regulatory factor 4. (IRF4) deficiency inherited by uniparental isodisomy. J Allergy Clin Immunol. (2018) 141:1924–7.e1918. 10.1016/j.jaci.2017.12.995 29408330

[43] Guérin A Kerner G Marr N Markle JG Fenollar F Wong N. IRF4 haploinsufficiency in a family with Whipple's disease. Elife. (2018) 7:e43229. 10.7554/eLife.32340.036 29537367 PMC5915175

[44] Taubenheim N von Hornung M Durandy A Warnatz K Corcoran L Peter HH. Defined blocks in terminal plasma cell differentiation of common variable immunodeficiency patients. J Immunol. (2005) 175:5498–503. 10.4049/jimmunol.175.8.5498 16210658

[45] Arjunaraja S Nosé BD Sukumar G Lott NM Dalgard CL Snow AL. Intrinsic plasma cell differentiation defects in B cell expansion with NF-κB and T cell anergy patient B cells. Front Immunol. (2017) 8:913. 10.3389/fimmu.2017.00913 28824638 PMC5539167

[46] Sundling C Forsell MN O'Dell S Feng Y Chakrabarti B Rao SS. Soluble HIV-1 Env trimers in adjuvant elicit potent and diverse functional B cell responses in primates. J Exp Med. (2010) 207:2003–17. 10.1084/jem.20100025 20679401 PMC2931166

[47] Banko Z Pozsgay J Szili D Toth M Gati T Nagy G. Induction and differentiation of IL-10-producing regulatory b cells from healthy blood donors and rheumatoid arthritis patients. J Immunol. (2017) 198:1512–20. 10.4049/jimmunol.1600218 28087671

[48] Halliley JL Tipton CM Liesveld J Rosenberg AF Darce J Gregoretti IV. Long-Lived Plasma Cells Are Contained within the CD19(-)CD38(hi)CD138(+) Subset in Human Bone Marrow. Immunity. (2015) 43:132–45. 10.1016/j.immuni.2015.06.016 26187412 PMC4680845

[49] Good KL Avery DT Tangye SG. Resting human memory B cells are intrinsically programmed for enhanced survival and responsiveness to diverse stimuli compared to naive B cells. J Immunol. (2009) 182:890–901. 10.4049/jimmunol.182.2.890 19124732

[50] Warnatz K Schlesier M. Flowcytometric phenotyping of common variable immunodeficiency. Cytometry B Clin Cytom. (2008) 74:261–71. 10.1002/cyto.b.20432 18561200

[51] Rösel AL Scheibenbogen C Schliesser U Sollwedel A Hoffmeister B Hanitsch L. Classification of common variable immunodeficiencies using flow cytometry and a memory B-cell functionality assay. J Allergy Clin Immunol. (2015) 135:198–208. 10.1016/j.jaci.2014.06.022 25112698

[52] Setz CS Hug E Khadour A Abdelrasoul H Bilal M Hobeika E. PI3K-Mediated Blimp-1 activation controls B cell selection and homeostasis. Cell Rep. (2018) 24:391–405. 10.1016/j.celrep.2018.06.035 29996100 PMC6057491

