# Supplementary figures and images for: Flow Cytometry-Based Protocols for the Analysis of Human Plasma Cell Differentiation
Source: Front Immunol. 2020 Sep 29;11:571321. doi: 10.3389/fimmu.2020.571321 (PMC7550473; doi:10.3389/fimmu.2020.571321)

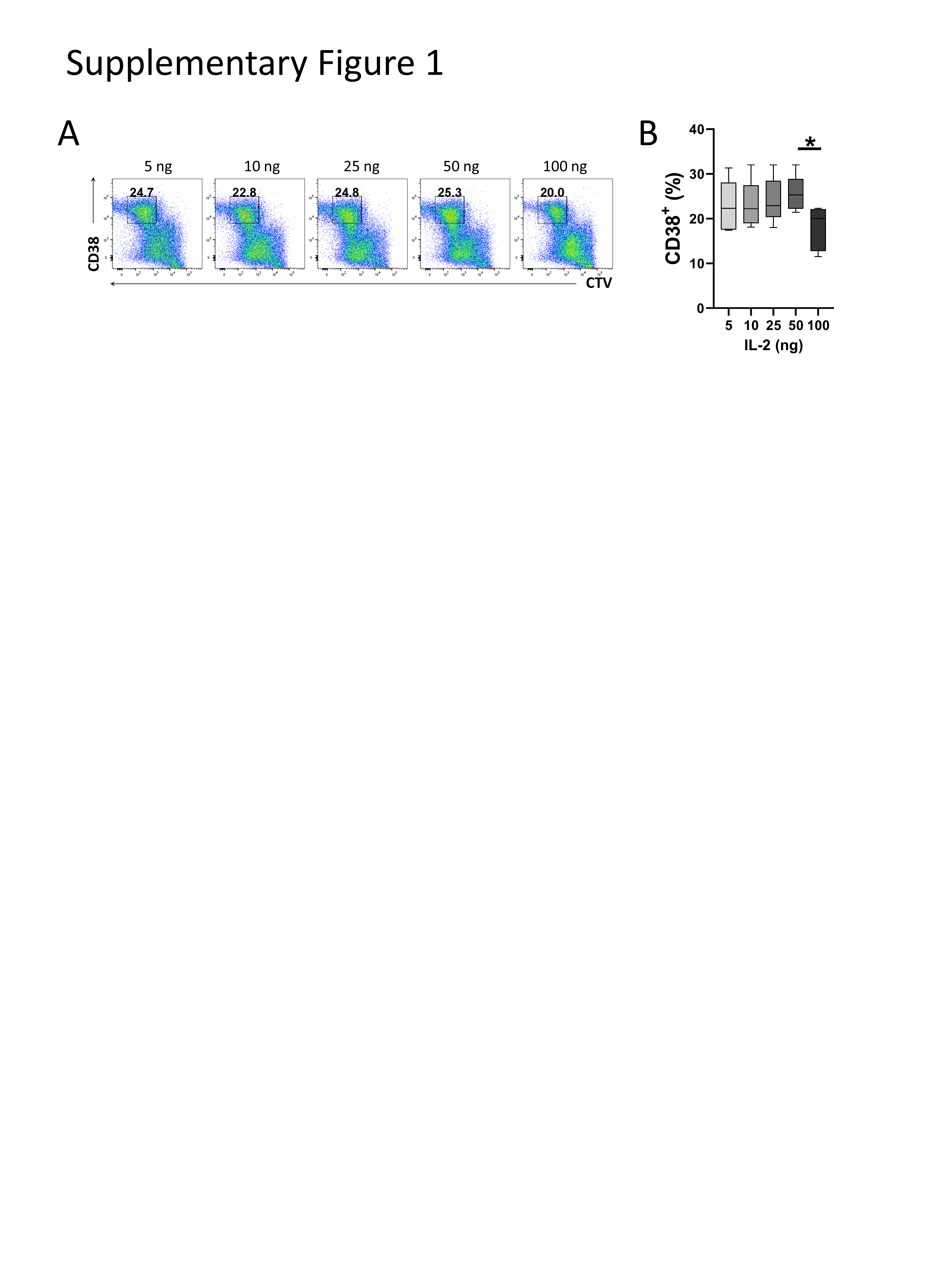

Supplement: Supplementary Figure 1 — Titration of IL-2. Human primary B cells were cultured with stimulation IV containing 5, 10, 25, 50, or 100 ng IL-2 with the same concentration used in the media for day 0–4 as well as day 4–6. Cells were pre-gated on FSC-A/FSC-W to identify singlets and on FSC-A/SSC-A to identify lymphocytes. (A) Representative flow cytometry plots showing gating strategy for CD38+ cells. Numbers adjacent to gates indicate cell frequencies. (B) Frequencies of CD38+ cells are summarized in a box plot with whiskers indicating the minimum and maximum values. Statistical significance was determined using Mann-Whitney U-test. Data are representative of two experiments with 5 donors. [file Image_1.TIF]

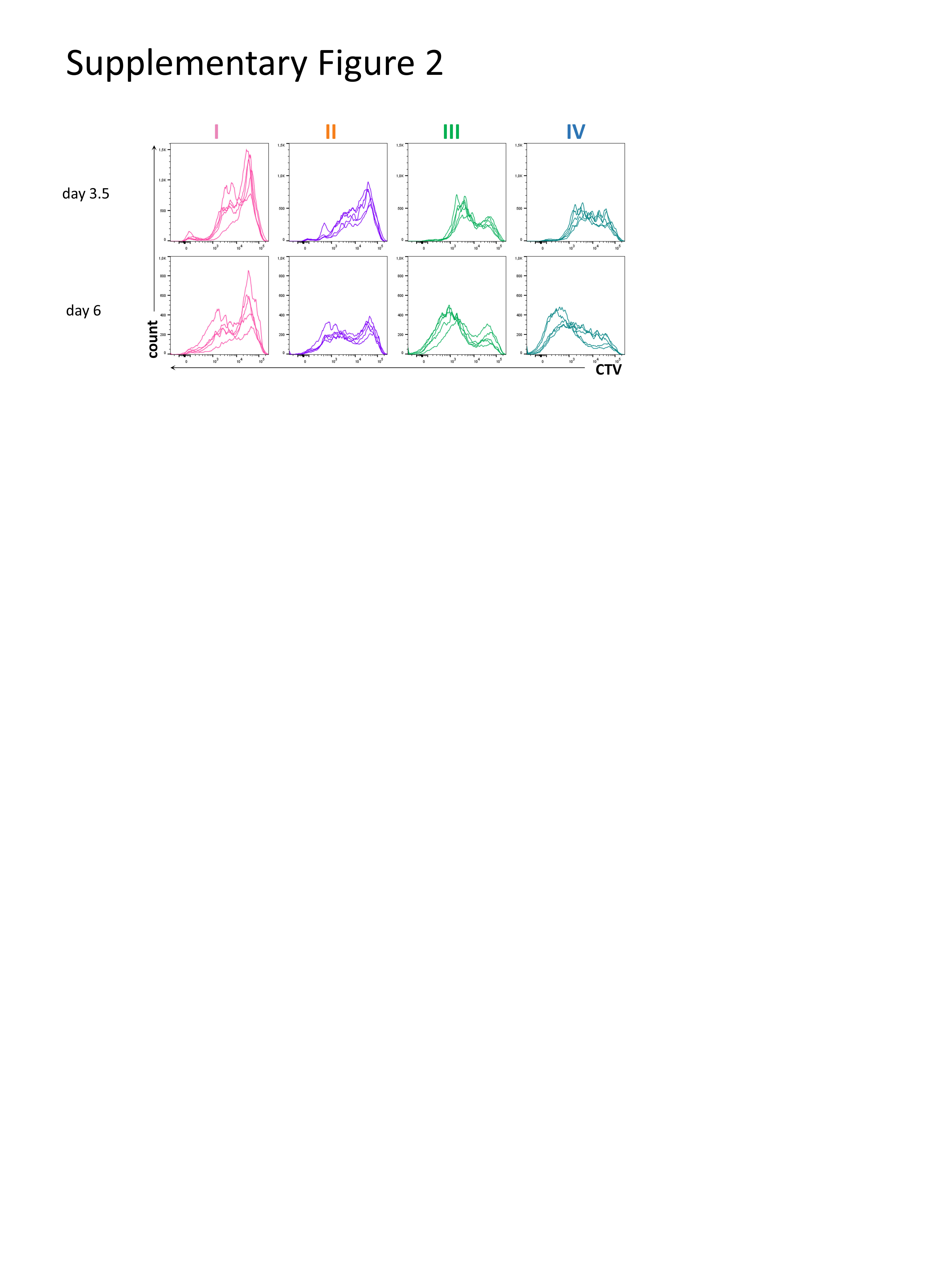

Supplement: Supplementary Figure 2 — Cellular divisions of stimulated B cells. Total B cells were isolated from healthy donor blood, labeled with CTV, stimulated under conditions described in Figure 1A, and analyzed at day 3.5 and day 6 by flow cytometry. Cells were pre-gated on FSC-A/FSC-W to identify singlets and on FSC-A/SSC-A to identify lymphocytes. Representative histogram overlays show cellular divisions from five donors based on CTV dilution. Data are representative of three independent repeats with 5 donors per experiment. [file Image_2.TIF]

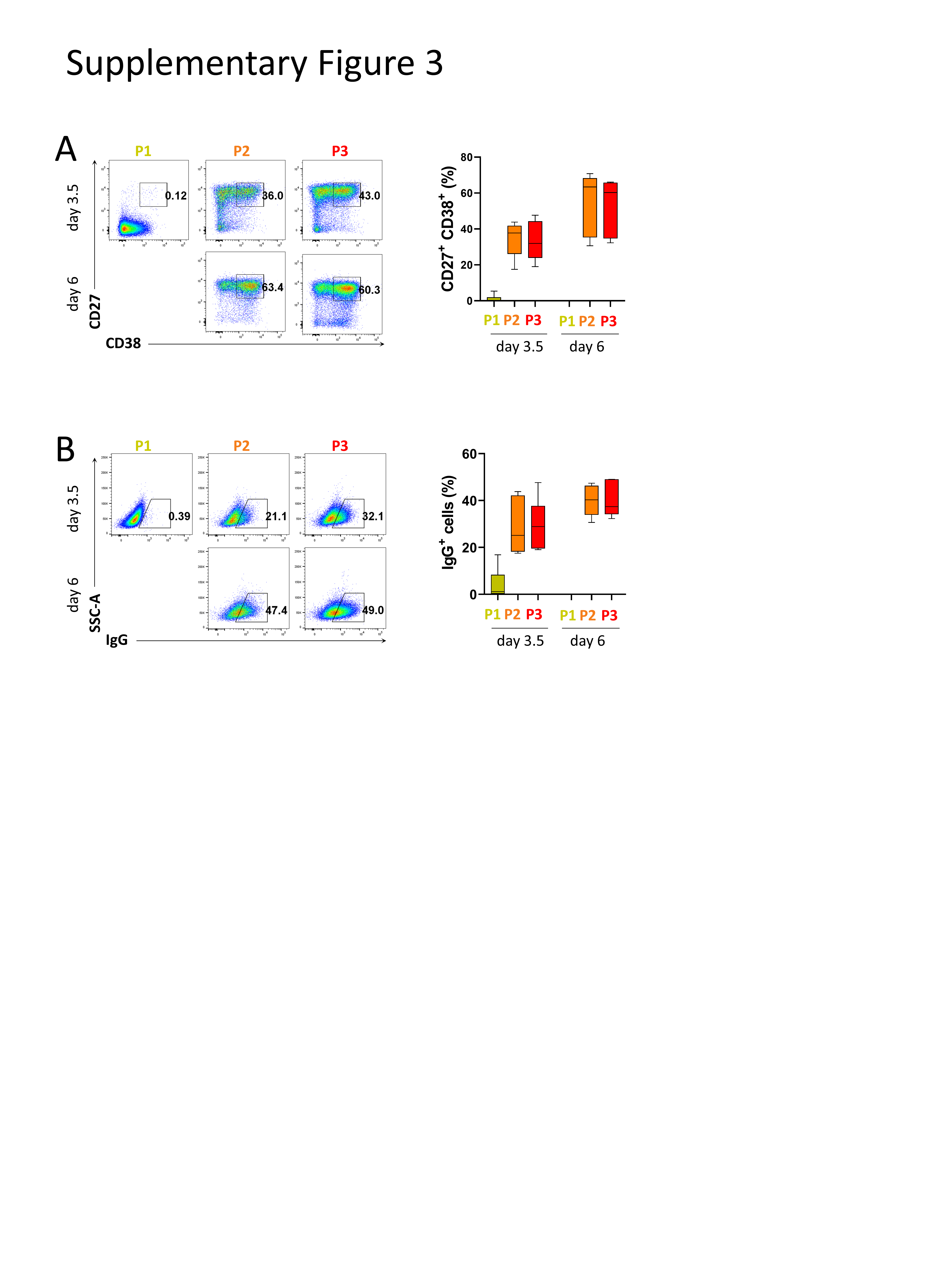

Supplement: Supplementary Figure 3 — Population P2 and P3 contain cells with a plasma blast/plasma cell phenotype. Total B cells were isolated from healthy donor blood, cultured with stimulation III, and analyzed at day 3.5 and day 6 by flow cytometry. Cells were pre-gated on FSC-A/FSC-W to identify singlets, on FSC-A/SSC-A to identify lymphocytes, on Live/Dead Green− to exclude dead cells, and on IRF4 and Pax5 expression to identify populations P1, P2, and P3. (A) Representative plots showing gating strategy for CD27+ CD38+ cells (left). Numbers adjacent to gates indicate cell frequencies. Frequencies of CD27+ CD38+ cells are summarized in a box plot with whiskers indicating the minimum and maximum values (right). (B) Representative plots showing gating strategy for intracellular IgG+ cells (left). Numbers adjacent to gates indicate cell frequencies. Frequencies of IgG+ cells are summarized in a box plot with whiskers indicating the minimum and maximum values (right). Data are pooled from two independent repeats with 3 donors per experiment. [file Image_3.TIF]

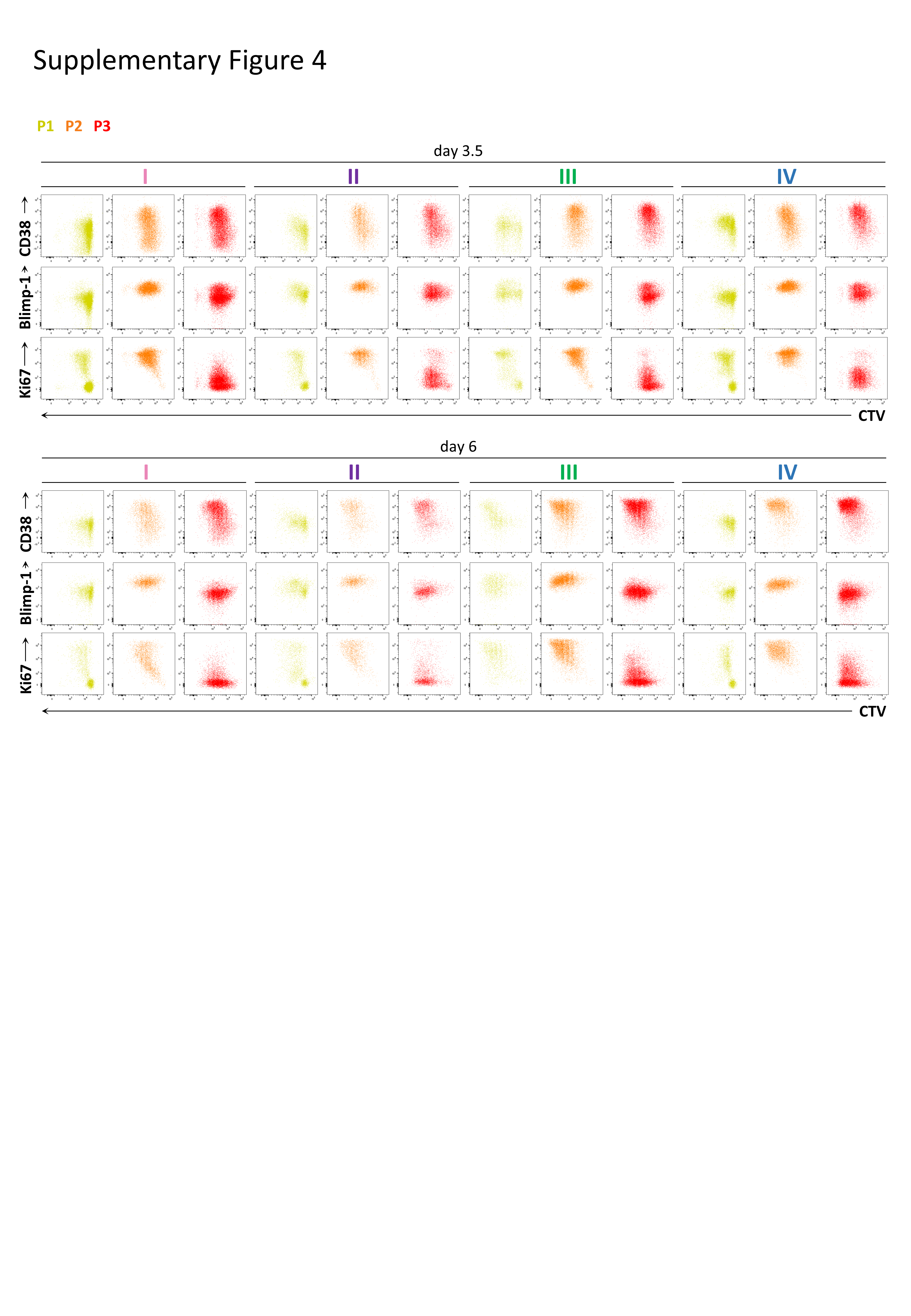

Supplement: Supplementary Figure 4 — Flow cytometric analysis of CD38, Blimp-1 and Ki67 expression in the P1, P2, and P3 populations. Total human B cells from healthy donors were stimulated under conditions described in Figure 1A and analyzed by flow cytometry at days 3.5 and 6. Cells were pre-gated on FSC-A/FSC-W to identify singlets, on FSC-A/SSC-A to identify lymphocytes, and on IRF4/ Pax5 expression to identify the P1, P2, and P3 populations. Representative plots showing CD38, Blimp-1 and Ki67 expression for the IRF4low Pax5hi (P1), IRF4hi Pax5lo (P2), and IRF4int Pax5lo (P3) populations. [file Image_4.tif]

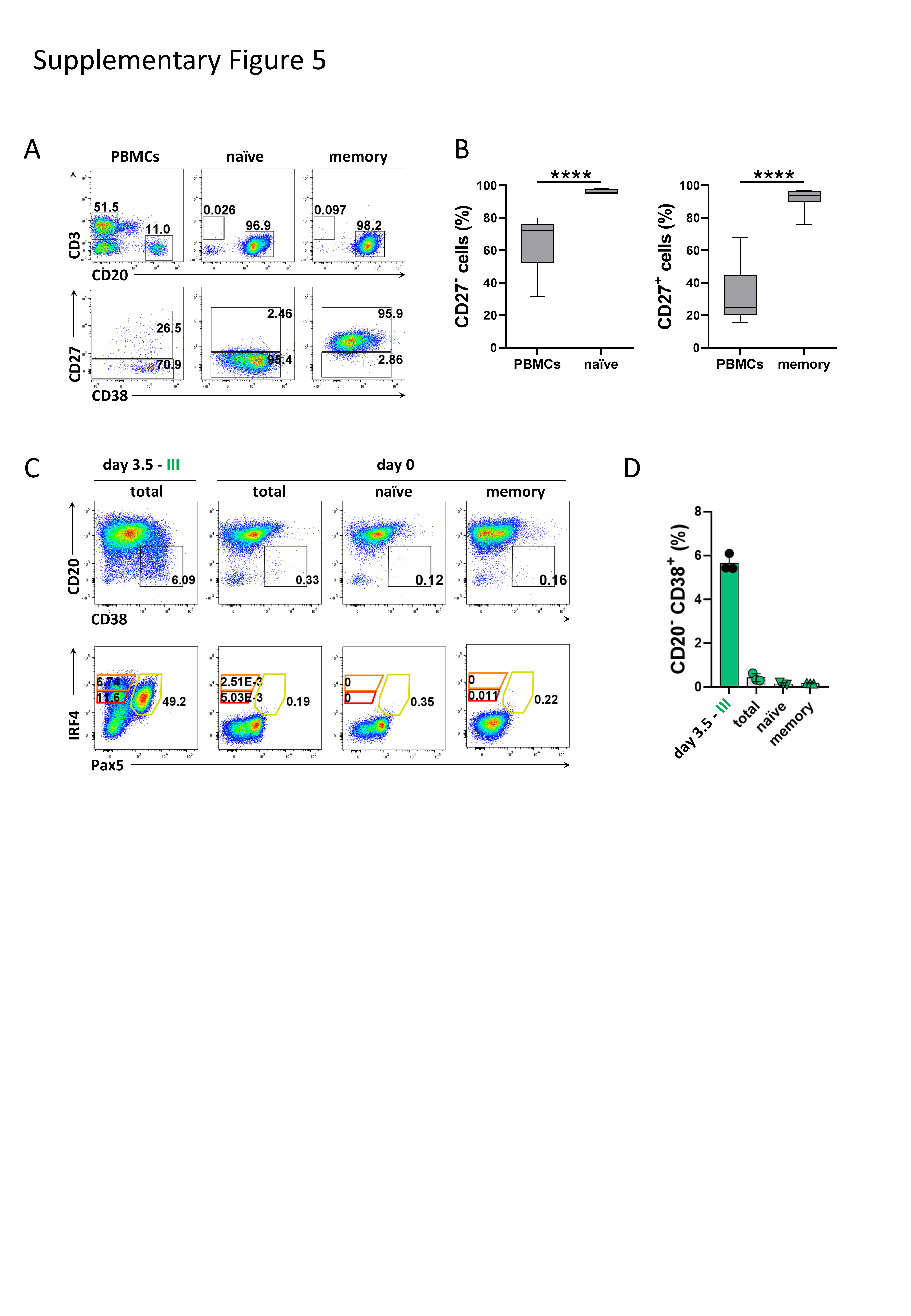

Supplement: Supplementary Figure 5 — Purity of naïve and memory B cell populations before and after enrichment. (A) Representative plots showing gating strategy for CD20+ CD38+ CD27− naïve and CD20+ CD38+ CD27+ memory B cell populations from fresh PBMCs and enriched populations. Cells were pre-gated on FSC-A/FSC-W to identify singlets and on FSC-A/SSC-A to identify lymphocytes. Numbers adjacent to gates indicate cell frequencies. (B) Frequencies of CD20+ CD38+ CD27− naïve and CD20+ CD38+ CD27+ memory B cell populations before and after enrichment. Data are representative of three independent repeats with 4-5 donors per experiment. (C) Representative plots showing gating strategy for CD20−CD38+ cells and populations P1, P2, and P3 based on IRF4 and Pax5 expression from total, memory, and naïve B cells immediately after enrichment. Cells cultured for 3.5 days with stimulation III are included as gating control. Cells were pre-gated on FSC-A/FSC-W to identify singlets, on FSC-A/SSC-A to identify lymphocytes, and on Live/Dead Green− to exclude dead cells. Numbers adjacent to gates indicate cell frequencies. (D) Frequencies of CD20−CD38+ cells. Data are representative of two independent repeats with 3 donors per experiment. [file Image_5.tif]

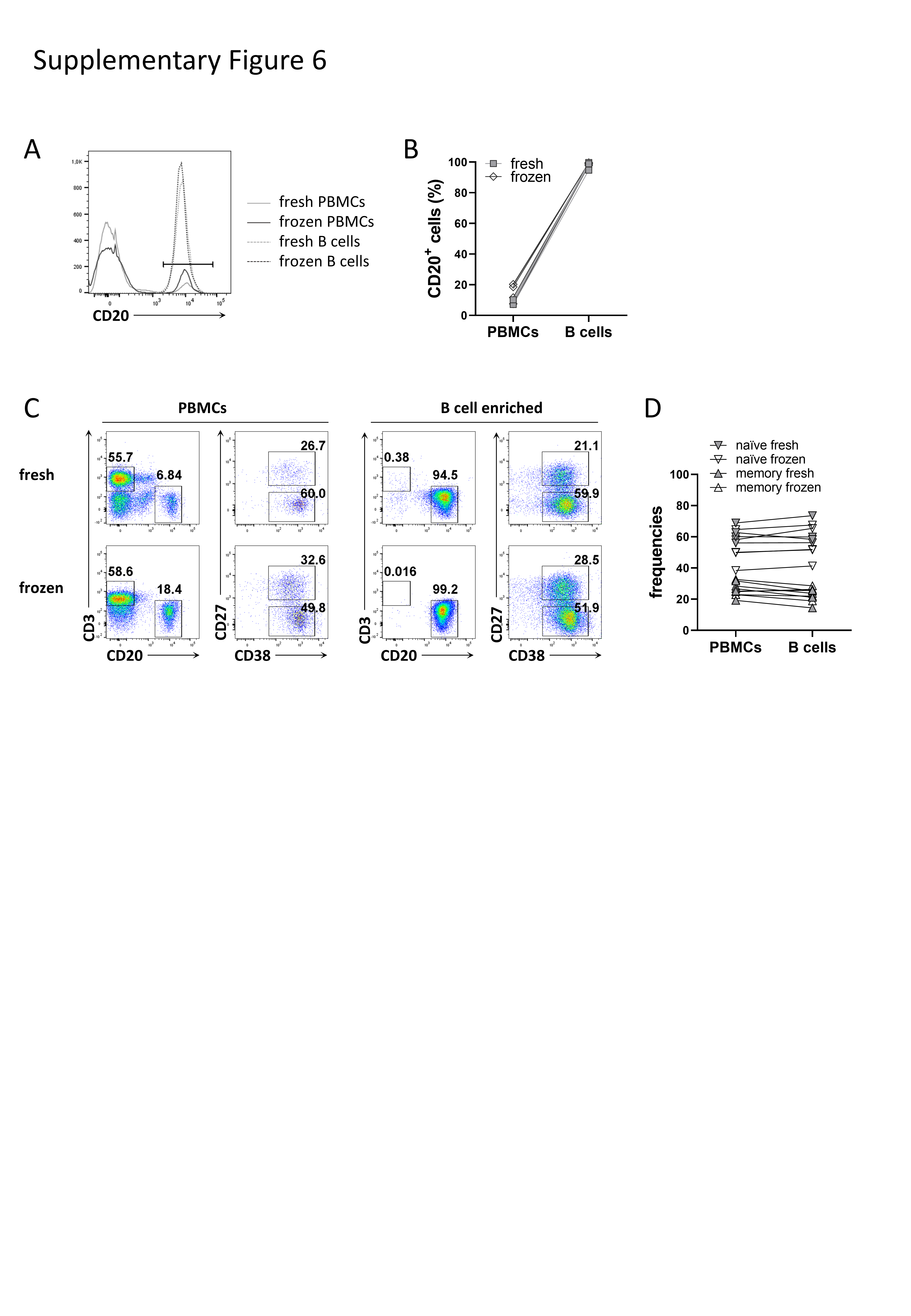

Supplement: Supplementary Figure 6 — Naïve and memory B cell populations in pre- and post-isolation samples from fresh PBMCs and frozen PBMCs. Cells were pre-gated on FSC-A/FSC-W to identify singlets and on FSC-A/SSC-A to identify lymphocytes. (A) Representative histogram showing gating strategy for the CD20+ population before and after isolation. (B) Frequencies of CD20+ B cells before and after isolation are shown for four samples from fresh PBMCs and four samples from frozen PBMCs. (C) Representative plots showing gating strategy for CD20+ CD38+ CD27− naïve and CD20+ CD38+ CD27+ memory B cell populations from fresh PBMCs (left panel) and frozen PBMCs (right panel). Numbers adjacent to gates represent the frequencies of cells gated on. (D) Graph showing frequencies of CD20+ CD38+ CD27− naïve and CD20+ CD38+ CD27+ memory B cell populations before and after isolation. Data are representative of three independent repeats with 4–5 donors per experiment. [file Image_6.tif]

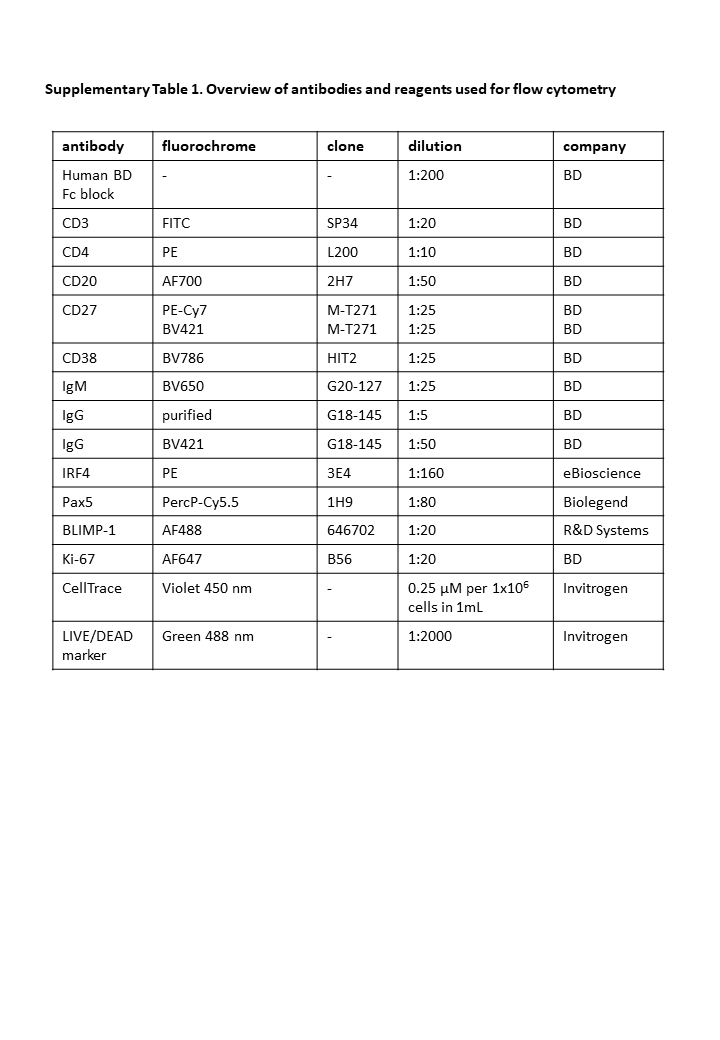

Supplement: Supplementary file 7 [file Image_7.TIF]
