# Supplementary material for: Flow Cytometry-Based Protocols for the Analysis of Human Plasma Cell Differentiation
Source: Front Immunol. 2020 Sep 29;11:571321. doi: 10.3389/fimmu.2020.571321 (PMC7550473; doi:10.3389/fimmu.2020.571321)
Supplement: Supplementary file 8 [file Data_Sheet_1.docx]

**Supplementary Protocols

1.1 Isolation of PBMCs from blood through density gradient separation using Ficoll Paque**1. Divide donor blood by adding 10-12.5 ml into 50 ml Falcon tubes containing 20 ml PBS (Sigma).
2. Use a 10 ml serological pipette to carefully underlay the blood with Ficoll Paque.
3. Gently top up with PBS to an end volume of 50 ml.
4. Spin for 20 minutes at 2000 rpm at room temperature using slow acceleration and deceleration, with brake off.
5. Collect and transfer the leukocyte fraction at the interphase between Ficoll Pacque and plasma to a new 50 ml Falcon tube.
6. Wash leukocyte fraction by adding PBS to the Falcon tube to 50 ml, spin for 10 minutes at 1750 rpm at room temperature, discard supernatant and disrupt pellet.
Repeat for a second wash.
7. Add 3 ml red blood cell lysis buffer (diluted 1:10 in ultrapure water) and incubate for 5 minutes at room temperature.
8. Wash by adding PBS to the Falcon tube to 50 ml, spin for 5 minutes at 1500 rpm at room temperature, discard supernatant, and disrupt pellet. Repeat for a second wash, this time spin for 10 minutes at 1250 rpm at room temperature.
9. (a – to continue directly with the B cell isolation) Resuspend and pool fractions from the same donor together in an end volume of 20 ml complete medium.
(b – to cryopreserve cells) Resuspend the cells at a concentration of 5x10^7^ cells per ml in freezing medium (FBS + 10% DMSO) for cryopreservation in Mr. Frosty freezing containers at -80°C. After freezing, transfer samples from Mr. Frosty to a regular cryobox and store at -80°C or in liquid nitrogen.
(c) Thaw samples at 37°C in a water bath, transfer the cells to 15 ml Falcon tube and carefully add pre-warmed complete medium up to 15 ml. Spin for 5 minutes at 1500 rpm at room temperature, discard supernatant and disrupt pellet. Repeat for a second wash with complete medium. Resuspend cells in 5 ml of complete medium and filter through a 70 µm cell strainer to remove debris.
10. Count cells.

**1.2 Isolation of B cells from PBMCs**1. Prepare cells at a concentration of 5x10^7^ cells per ml in isolation buffer (PBS containing 2% FBS and 1 mM EDTA). For isolation from 2x10^8^ PBMCs, cells are resuspended in 4 ml isolation buffer and transferred to a flat-bottom 6-well plate.
For a yield of 5x10^5^ B cells after enrichment, use a minimum of 5x10^7^ PBMCs for the isolation procedure (based on normal B cell frequencies in blood).
2. Add EasySep Human B Cell Enrichment Cocktail at a concentration of 50 µl/ml to samples and incubate for 10 minutes at RT.
3. Add EasySep D Magnetic Particles at a concentration of 75 µl/ml to samples and incubate for 5 minutes at RT.
4. Top up volume to 6 ml with isolation buffer, place plate on the EasyPlate EasySep magnet, and incubate for 5 minutes.
5. Transfer supernatant to a 15 ml Falcon tube, top up to 15 ml with PBS, and spin down at 1250 rpm for 5 minutes. Discard supernatant, disrupt cell pellet, and top up to 15 ml with PBS. Spin down at 1250 rpm for 5 minutes, discard supernatant, discard cell pellet, and resuspend in PBS.
6. Count cells. A minimum amount of 2.5x10^5^ cells is required for assessing plasma cell differentiation per time point.

**1.3 CellTrace Violet labelling**
1. For cell labelling prepare 1 ml of CTV in PBS per 1x10^6^ cells; use CTV at a dilution of 1:4000 (corresponding to 0.25 µM per 1x10^6^ cells).
2. Transfer desired number of cells for labelling (e.g. 1x10^6^) to a new 15 ml Falcon tube, top up to 15 ml with PBS, spin down at 1250 rpm for 5 minutes. Discard supernatant and disrupt pellet.
3. Add 1 ml of 0.25 µM CTV-PBS per 1x10^6^ cells, resuspend cells gently in solution. Incubate cells for 20 minutes at 37°C incubator.
4. Top up to 15 ml with pre-warmed complete medium. Incubate for 5 minutes at 37°C.
5. Spin down cells at 1250 rpm for 5 minutes. Discard supernatant and disrupt pellet.
6. Resuspend cells at a concentration of 5x10^5^ cells per ml in complete medium.
7. Keep cells on ice until the plate is prepared for seeding.

**2. *In vitro* B cell stimulation**1. Prepare medium for stimulation: prepare 500 µl of medium per well.
2. Add anti-IgM F(ab’)_2_ fragments (10 μg/mL), CpG (5 μg/mL), sCD40L (2 μg/mL), and recombinant human IL-21 (100 ng/mL) to the medium.
3. Add 500 µl of stimulation medium per well. (NB: use inner wells on the plate for stimulation, fill outer wells on the plate with PBS to prevent evaporation of samples).
4. Add 500 µl of cells per well. Resuspend gently. Each well now contains 2.5x10^5^ cells in 1 ml complete medium with 5 μg/mL anti-IgM F(ab’)_2_ fragments, 2.5 μg/mL CpG, 1 μg/mL sCD40L, and 50 ng/mL recombinant human IL-21.
5. Culture cells at 37°C for 3.5 and 6 days.

**3. Flow cytometry staining**
1. Transfer cells to 5 ml polystyrene tubes, wash them with 3 ml of FACS buffer, spin down at 1250 rpm for 5 minutes. Discard supernatant and disrupt pellet.
2. Add 50 µl of FACS buffer containing Fc block to each sample. Incubate for 20 minutes on ice in dark.
3. Wash: add 3 ml FACS buffer to each sample and spin down at 1250 rpm for 5 minutes. Discard supernatant and disrupt pellet.
4. Add 50 µl surface panel antibodies (CD20, CD38) diluted in FACS buffer to each sample. Incubate for 20 minutes on ice in dark.
5. Wash: add 3 ml FACS buffer to each sample and spin down at 1250 rpm for 5 minutes. Discard supernatant and disrupt pellet.
6. Fixation/permeabilization: add 100 µl of fixation/permeabilization buffer (BD Transcription Factor Buffer set; fix/perm buffer diluted 1:3 in diluent buffer) per sample and resuspend, (a) incubate for 50 minutes on ice in dark or (b) samples can be stored in fixation/permeabilization buffer at 4°C and protected from light.
7. Wash: add 1 ml of permeabilization/wash buffer (BD Transcription Factor Buffer set; perm/wash buffer diluted 1:4 in ultrapure water) per sample and spin down at 1600 rpm for 6 minutes. Discard supernatant and disrupt pellet.
8. Resuspend samples in 50 µl of intracellular panel antibodies (Pax5, IRF4, Blimp-1, Ki67) diluted in perm/wash buffer to each sample and incubate for 6 h on ice in dark.
9. Wash: add 1 ml of permeabilization/wash buffer per sample (BD Transcription Factor Buffer set; perm/wash buffer diluted 1:4 in ultrapure water) and spin down at 1600 rpm for 6 minutes. Discard supernatant and disrupt pellet.
10. Resuspend samples in 200 µl FACS buffer.
11. Acquire samples on the flow cytometer.
 **4.1 Collection of supernatant for ELISA**1. Transfer cells to 5 ml polystyrene tubes and spin at 1250 rpm for 5 minutes.
2. Separate supernatant into 1.5 ml Eppendorf tubes.
3. Store supernatant at -20°C if not used directly.

**4.2 Detection of IgG by ELISA**1. Coating of plates: add 100 µl of goat anti-human unlabelled IgG diluted 1:1000 in PBS to each well. Cover plates with plate seal to prevent evaporation and incubate overnight at 4°C.
2. Wash plates six times with washing buffer (PBS+0.05% Tween20), discard residual wash buffer, and dry-tap plates on tissue paper.
3. Add 200 µl of blocking buffer and incubate for 1 h at RT.
4. Prepare plates for addition of supernatant: discard blocking buffer and add 100 µl of fresh blocking buffer to each well.
5. Add 50 μl of culture supernatant to the first well, followed by 3-fold serial dilution in following wells, and incubate the plate for 2 hs at room temperature.
6. Wash plates six times with washing buffer, discard residual wash buffer, and dry-tap plates on tissue paper.
7. Add 100 µl of HRP-coupled goat anti-human IgG diluted 1:1000 in PBS per well and incubate for 1.5 h at RT.
8. Wash plates six times with washing buffer, discard residual wash buffer, and dry-tap plates on tissue paper.
9. Develop the assay by adding 100 µl TMB substrate (KLP) per well and incubate for 3-5 minutes protected from light.
10. Stop the reaction by adding 100 µl 1 M H_2_SO_4_ per well.
11. Read the OD values at 450 nm using a spectrophotometer.
